# Effect of root canal filling techniques and materials on endodontic treatment outcomes: a systematic review and meta-analysis

**DOI:** 10.1038/s41598-026-37936-7

**Published:** 2026-03-23

**Authors:** Ahmed Mushtaq, Sura Alsanafi, Firas Elmsmari, José A. González, Marc Garcia-Font, Francesc Abella Sans, Kelvin I. Afrashtehfar, Paul V. Abbott

**Affiliations:** 1 Clinical Sciences Department, College of Dentistry, Ajman University, P.O. Box 346, Ajman, UAE; 2 Department of Endodontics, Faculty of Dentistry, Universitat Internacional de Catalunya (UIC), Barcelona, 08195 Spain; 3 Department of Reconstructive Dentistry and Gerodontology, School of Dental Medicine, Universität Bern, Bern, 3010 Switzerland; 4 Consultant Implantologist private practice, Oral Implantology Research Institute (OIRI), Dubai, UAE; 5 Specialist Prosthodontist private practice, Abbotsford, BC Canada; 6 UWA Dental School, The University of Western Australia, Perth, WA Australia

**Keywords:** Apical periodontitis, Bioceramics, Carrier-based techniques, Clinical outcomes, Cold lateral condensation, Dental materials, Dental restoration failure, Endodontic retreatment, Endodontics, Evidence-based dentistry, Follow-up studies, Gutta-percha, Longitudinal studies, Meta-analysis, Prognosis, Radiographic image interpretation, dental, Root canal filling materials, Root canal filling techniques, Root canal therapy, Systematic review, Success rate, Thermoplastic techniques, Tooth pulp pathology, Treatment outcome, Outcomes research, Endodontics, Root canal treatment

## Abstract

Root canal filling is a critical determinant of endodontic treatment success, yet limited evidence exists regarding the superiority of specific techniques or materials over others. This systematic review and meta-analysis compared the clinical and radiographic outcomes of different root canal obturation techniques and materials in primary treatments and retreatments. We systematically searched PubMed, Cochrane Library, and ScienceDirect up to the last week of November 2025 following PRISMA guidelines. Studies were included if they reported primary or retreatment outcomes with at least 6 months of follow-up, < 25% loss to follow-up, and explicit documentation of obturation techniques and clinical/radiographic success. Risk of bias was assessed with ROBINS-I (cohort studies) and RoB 2.0 (randomized studies), and the overall evidence certainty was rated using GRADE. Success rates were compared across techniques using mixed-effects meta-regression, estimating differences in outcomes (beta coefficients) with 95% confidence intervals and accounting for between-study heterogeneity. Eighty-four studies (11,965 samples) met all inclusion criteria. In primary treatments, overall success rates were 87.1% at 6 months and 87.2% at 12 months, without significant differences among techniques. At 24 months, success increased to 92.0%; cold lateral condensation (CLC) (difference: 5.0%, *p* = 0.021) and carrier-based (CB) techniques (difference: 7.5%, *p* = 0.011) showed higher success than single-cone (SC). Beyond 3 years, success decreased to 84.9%, with no statistically significant differences among warm vertical compaction (WVC), warm lateral compaction (WLC), CLC, SC, and CB techniques. In retreatments, success rates were 92.9% at 6 months, 77.0% at 12 months, and 83.5% at 24 months. At 12 months, SC showed a marginally higher success than CLC (*p* = 0.045), while at 24 months, CB significantly outperformed WVC (*p* = 0.004). Beyond 3 years, success decreased to 73.7%, with no significant differences between CLC and CB techniques. Overall, primary treatments presented higher success than retreatments. At 24 months, CLC and CB techniques showed modest advantages, which were not maintained at longer follow-ups. Success appears multifactorial, with operator expertise and case selection having greater impact than obturation technique. The overall certainty of evidence remained low to very low, particularly for long-term outcomes. Bioceramic sealers used with SC techniques have not demonstrated clear clinical benefits or improved retreatment outcomes. High heterogeneity and risk of bias suggest a need for well-powered, multicenter, long-term randomized trials with standardized outcome reporting to establish clinical standards (PROSPERO registration: CRD42024524608).

**Supplementary Information:**

The online version contains supplementary material available at 10.1038/s41598-026-37936-7.

## Introduction

Root canal filling represents a critical phase in endodontic treatment, aimed at sealing the canal system, preventing reinfection, and supporting long-term success. The quality of root canal filling, including its apical sealing, homogeneity, and adaptation, has been identified as an important factor influencing the prognosis of treated teeth^1–3^. An optimal apical seal is critical to prevent microbial leakage and minimize post-treatment disease.

Historically, root canal fillings have been completed using a combination of core materials and sealers. The cold lateral condensation (CLC) technique, recognized as the classical technique, remains the most widely used due to its predictable control of filling length and cost-effectiveness^4^. However, its limited ability to achieve three-dimensional sealing, particularly in irregular canal spaces, led to the introduction of warm vertical compaction (WVC) technique by Schilder in 1967, which improved material adaptation to the canal system, particularly its apical and lateral irregularities^5^. Since then, more advanced thermoplastic techniques have evolved, such as continuous wave condensation, injection-molded gutta-percha, and carrier-based (CB) techniques, each presenting distinct advantages and limitations based on specific clinical scenario^6,7^.

Despite these advancements, the impact of obturation technique on treatment outcomes remains unclear. While early studies focused primarily on radiographic parameters such as the extent of the root filling relative to the apical foramen^8–10^, recent evidence has reinforced the critical role of achieving a well-sealed apical third to ensure treatment success^11,12^. However, most of the existing literature focused on the radiographic quality of root canal fillings, with little emphasis on direct comparisons of success rates between different techniques and materials. In recent years, bioceramic sealers have gained popularity due to their bioactivity and superior sealing potential. However, high-quality comparative studies directly evaluating their performance against traditional sealers in both primary root canal treatments (RCTs) and retreatment cases (re-RCTs) remain limited. This gap in evidence is significant, as prior filling materials can complicate retreatment procedures and influence outcomes.

With the increasing use of single-cone (SC) techniques with bioceramic sealers, alongside established methods such as CLC, WVC, and CB, there is a need for rigorous clinical comparisons. Evaluating success rates across these methods, particularly in retreatment cases, could offer clinicians evidence-based guidance for optimizing treatment protocols. Therefore, this systematic review and meta-analysis compared the clinical and radiographic success rates of root canal filling techniques and materials, aiming to support standardized and effective endodontic care.

## Materials and methods

### Protocol and registration

This systematic review and meta-analysis adhered to the Preferred Reporting Items for Systematic Reviews and Meta-Analyses (PRISMA) guidelines^13^ to promote comprehensive and transparent reporting, and follows the methodological approach of a recent publication^14^. The protocol was registered in the PROSPERO international prospective database (Registration ID: CRD42024524608), which provides a publicly accessible record of the study plan and methodology.

### Focused research question

Using the PICOTSS framework, this systematic review addressed the following question: In human permanent teeth undergoing RCT or re-RCT (P), do different obturation techniques and/or materials (I), compared with alternative ones (C), influence endodontic treatment outcomes (O) at a minimum follow-up of six months (T), based on evidence from clinical studies conducted in dental or academic settings (S, S)?

Endodontic treatment outcomes were defined a priori as clinical and radiographic success. Prespecified subgroup analyses included treatment type (RCT and re-RCT), obturation technique (CLC, WVC, WLC, SC, CB), follow-up duration, and study design.

### Eligibility criteria

This systematic review included randomized controlled trials and cohort studies published in any language up to November 30th, 2025. Studies were included if they involved permanent teeth, described the root canal filling technique and material, had a minimum follow-up of six months, and reported a dropout rate ≤ 25%, aligning with previous meta-analysis in Endodontology^107^. Outcomes had to be assessed using defined clinical and radiographic criteria.

Studies were excluded if they were case reports, case series, review articles, or animal and in vitro studies. Research involving primary teeth, studies with incomplete information on techniques or materials, or those without clear radiographic and clinical outcomes were also excluded.

### Search strategy

A comprehensive electronic search was performed independently by two authors across three major databases: PubMed (National Center for Biotechnology Information), Cochrane Library (John Wiley & Sons, Ltd), and ScienceDirect (Elsevier). The search was conducted without language or publication date restrictions to ensure the inclusion of all relevant studies.

To improve the completeness of the search, grey literature was explored using the following sources: CADTH’s Grey Matters (https://greymatters.cadth.ca/), The European database on medical devices (EUDAMED) (https://ec.europa.eu/tools/eudamed/#/screen/home), and The New York Academy of Medicine Library (https://catalog.nyam.org/cgi-bin/koha/opac-search.pl). A manual search was also conducted to review relevant journals, including the *International Endodontic Journal*,* Journal of Endodontics*,* Clinical Oral Investigations*,* European Endodontic Journal*,* Scientific Reports*, and the *Australian Endodontic Journal*, bibliographies of included studies, and conference proceedings to capture any missed publications or unpublished data.

The search strategy utilized a combination of MeSH terms and relevant keywords, including “root canal treatment,” “endodontic therapy,” “prognosis,” “outcome,” “root canal obturation,” and “root canal retreatment,” combined using Boolean operators such as ‘AND’ to narrow the search and ‘OR’ to include synonymous terms. The full applied search strategy for PubMed/Medline is presented in Table 1.

**Table 1:** Applied search strategy in PubMed/Medline electronic database.

Field of exploration	Combination of keywords and terms
*endodontics AND prognosis*	(“endodontal“[All Fields] OR “endodontic“[All Fields] OR “endodontical“[All Fields] OR “endodontically“[All Fields] OR “endodontics“[MeSH Terms] OR “endodontics“[All Fields]) AND (“prognosis“[MeSH Terms] OR “prognosis“[All Fields] OR “prognoses“[All Fields])
*endodontics AND outcome*	(“endodontal“[All Fields] OR “endodontic“[All Fields] OR “endodontical“[All Fields] OR “endodontically“[All Fields] OR “endodontics“[MeSH Terms] OR “endodontics“[All Fields]) AND (“outcome“[MeSH Terms] OR “outcome“[All Fields] OR “outcomes“[All Fields])
*root canal obturation*	“root canal obturation“[MeSH Terms] OR (“root canal obturation“[All Fields])
*root canal obturation AND prognosis*	(“root canal obturation“[MeSH Terms] OR “root canal obturation“[All Fields]) AND (“prognosis“[MeSH Terms] OR “prognosis“[All Fields] OR “prognoses“[All Fields])
*root canal obturation AND outcome*	(“root canal obturation“[MeSH Terms] OR “root canal obturation“[All Fields]) AND (“outcome“[MeSH Terms] OR “outcome“[All Fields] OR “outcomes“[All Fields])
*root canal AND outcome*	(“dental pulp cavity“[MeSH Terms] OR “dental pulp cavity“[All Fields] OR (“root canal“[All Fields])) AND (“treatment outcome“[MeSH Terms] OR “treatment outcome“[All Fields])
*root canal treatment prognosis*	(“dental pulp cavity“[MeSH Terms] OR “dental pulp cavity“[All Fields] OR (“root canal“[All Fields])) AND (“treatment“[MeSH Terms] OR “treatments“[All Fields] OR “therapy“[MeSH Terms]) AND (“prognosis“[MeSH Terms] OR “prognosis“[All Fields] OR “prognoses“[All Fields])
*root canal retreatment prognosis*	(“dental pulp cavity“[MeSH Terms] OR “dental pulp cavity“[All Fields] OR (“root canal“[All Fields])) AND (“retreatment“[MeSH Terms] OR “retreatment“[All Fields] OR “retreating“[All Fields]) AND (“prognosis“[MeSH Terms] OR “prognosis“[All Fields] OR “prognoses“[All Fields])
*root canal treatment outcome*	(“dental pulp cavity“[MeSH Terms] OR “dental pulp cavity“[All Fields] OR (“root canal“[All Fields])) AND (“retreatment“[MeSH Terms] OR “retreatment“[All Fields] OR “retreating“[All Fields]) AND (“outcome“[MeSH Terms] OR “outcome“[All Fields] OR “outcomes“[All Fields])
*root canal retreatment outcome*	(“dental pulp cavity“[MeSH Terms] OR “dental pulp cavity“[All Fields] OR (“root canal“[All Fields])) AND (“retreatment“[MeSH Terms] OR “retreatment“[All Fields] OR “retreating“[All Fields]) AND (“outcome“[MeSH Terms] OR “outcome“[All Fields] OR “outcomes“[All Fields])
*endodontic retreatment*	(“endodontal“[All Fields] OR “endodontic“[All Fields] OR “endodontical“[All Fields] OR “endodontically“[All Fields] OR “endodontics“[MeSH Terms] OR “endodontics“[All Fields]) AND (“retreatment“[MeSH Terms] OR “retreatment“[All Fields] OR “retreating“[All Fields])
endodont* AND retreatment	“endodont*“[All Fields] AND (“retreatment“[MeSH Terms] OR “retreatment“[All Fields] OR “retreating“[All Fields])
*root canal retreatment*	(“dental pulp cavity“[MeSH Terms] OR “dental pulp cavity“[All Fields] OR (“root canal“[All Fields])) AND (“retreatment“[MeSH Terms] OR “retreatment“[All Fields] OR “retreating“[All Fields])

### Screening and study selection

Initial screening was performed from June 1 to August 14, 2023. The search was subsequently updated, and the final screening was completed on November 30, 2025, to include the most recent eligible studies. Duplicate records were removed prior to title and abstract screening. Study selection was independently performed by two reviewers (A.M. and S.A.). Any disagreements were resolved by consensus, and when necessary, a third reviewer (F.E. or K.I.A.) acted as an arbitrator.

### Data extraction

After reviewing the titles and abstracts of the search results, studies that potentially met the inclusion criteria were subjected to full-text evaluation. Data extraction followed a predefined standardized collection form to ensure consistency and accuracy. Extracted information included study details such as authors, year of publication, and study design (randomized or cohort), along with sample characteristics including sample size, patient age, gender, and pulp/periapical status. Treatment-specific details were recorded, including the type of treatment (primary root canal treatment or retreatment), root canal filling techniques, materials, cement type, instrumentation technique, and irrigants used. Additional clinical variables extracted comprised tooth type, rubber dam utilization, operator skill, number of treatment sessions, and microscope use. Follow-up periods were classified into four distinct time points: 6 months, 12 months, 24 months, and more than 3 years.

Outcomes were assessed based on radiographic evaluations and resolution of clinical signs and symptoms, with data extracted for all reported follow-up periods to ensure a comprehensive analysis. Any disagreements between the two independent reviewers during the data extraction process were resolved through discussion. If consensus could not be reached, a third reviewer (F.E. or K.I.A.) served as an impartial arbitrator to finalize decisions.

### Quality assessment

To evaluate the risk of bias, two independent reviewers (A.M. and S.A.) conducted quality assessments for all included studies. For randomized studies, the revised Cochrane RoB2 tool^15^ was employed, addressing five key domains: randomization process, deviations from intended interventions, missing outcome data, outcome measurement, and reporting bias. For non-randomized studies, the ROBINS-I tool^16^ was utilized, assessing bias across seven domains including selection bias, performance bias, attrition bias, and reporting bias. Disagreements were resolved by discussion; if unresolved, a third reviewer (F.E. or K.I.A.) acted as an impartial arbitrator. The assessments were performed using RevMan 5.4 software (Cochrane Collaboration) to ensure systematic organization and visualization of results.

The overall certainty of evidence was evaluated using the GRADE system, which accounts for risk of bias, inconsistency, imprecision, and overall confidence in the evidence. This systematic approach allowed for a comprehensive evaluation of study quality and evidence strength.

### Statistical analysis

A comprehensive meta-analysis was conducted using a mixed-effects model, with root canal filling techniques serving as the moderator variable. To account for heterogeneity among studies, a restricted maximum likelihood estimator was applied. The overall effect size was presented using forest plots with 95% confidence intervals to illustrate the raw rate of successful outcomes across the included studies. Heterogeneity between studies was evaluated using Cochran’s Q test and quantified through the *I*² index, which represents the proportion of variability attributable to between-study differences. A funnel plot analysis was performed to visually inspect the potential presence of publication bias, while Egger’s test was used to statistically assess its significance. A significance level of α = 0.05 was applied to all statistical tests.

Subgroup analyses were performed to explore factors contributing to heterogeneity, including root canal filling techniques, material types, and moderating factors such as patient age, gender, tooth type, anesthetic solution, and treatment type (primary or retreatment). A meta-regression analysis was performed using a mixed-effects model to quantify the influence of these covariates on treatment outcomes. Results were reported as beta coefficients (β) with 95% confidence intervals and Z statistics, facilitating a clearer understanding of variable relationships. All statistical analyses were conducted using R software (version 4.3.1) (R Core Team, 2023), a robust statistical computing platform widely used in meta-analyses.

## Results

### Study selection

**Fig. 1:**
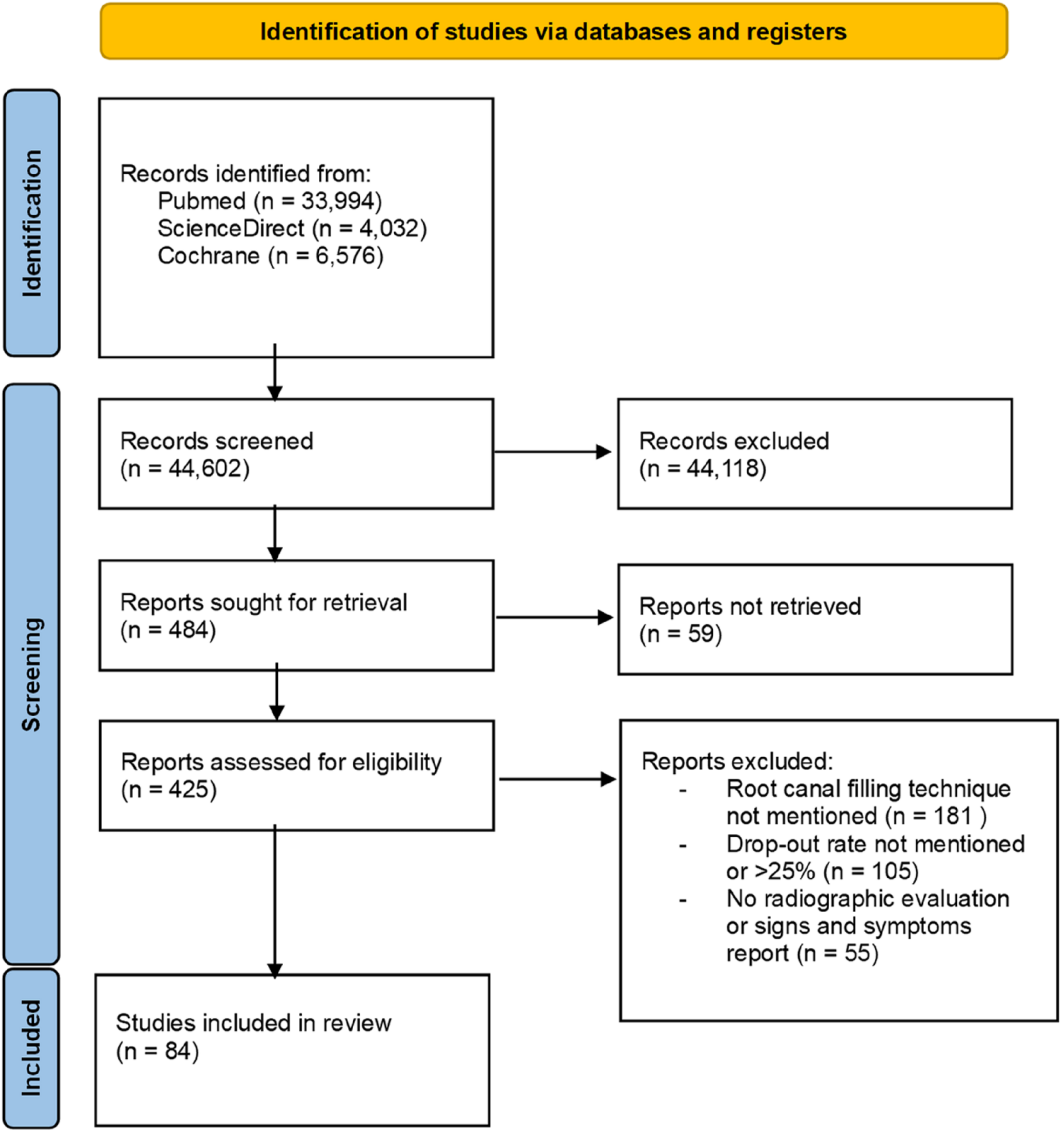
PRISMA flow diagram: visual representation of the study selection process.

The study selection followed PRISMA guidelines (Fig. 1). From 44,602 initial records, 484 full texts were assessed after duplicate removal and title/abstract screening. Of these, 341 were excluded due to ineligible design, insufficient follow-up, or incomplete outcomes (Suppl. Table 1). A total of 84 studies met inclusion criteria and were included in qualitative and quantitative analyses (Table 2).

**Table 2:** Characteristics of the included studies.

Follow up period/recall rate	Core material & cement/sealer	Root canal filling technique	Treatment type	Total samples	Study design	Author (year)
6 Months/78%	GP/Glass ionomer	CLC	Both	486	Prospective	Friedman et al. (1995)^17^
6 Months/100%	GP/Resin based	CLC TH	Primary	100	Prospective	Mohan et al. (2009)^18^
6 Months/100%	CaOH	AP	Primary	22	Randomized	Bezgin et al. (2012)^19^
6 Months/86%	GP/ZOE	CLC	Primary	36	Randomized	Martins et al. (2013)^20^
6 Months/100%	GP/AH Plus	CLC	Primary	70	Randomized	Ifeoma et al. (2015)^21^
6 months/80%	GP/AH Plus	CLC	Primary	60	Randomized	Kist et al. (2017)^22^
6 months/75%	GP/AH Plus	CLC	Primary	120	Randomized	Figueiredo et al. (2020)^23^
6 Months/98%	GP/AH Plus	CLC CB SC	Primary	40	Prospective	Shaheed et al. (2020)^24^
6 Months/75%	GP/AH Plus + BC	WVC	Both	100	Randomized	Kim et al. (2022)^25^
6 Months/100%	GP/ZOE	SC	Primary	206	Retrospective	Silva et al. (2023)^26^
6 Months/84%	GP/AH Plus + BC	WVC	Primary	76	Randomized	Hu et al. (2023)^27^
12 Months/100%	GP/Resin based	CLC	Retreatment	18	Randomized	Danin et al. (1996)^28^
12 Months/100%	GP/Sealer not mentioned	TH	Primary	131	Prospective	Tani-Ishii et al. (2003)^29^
12 Months/100%	GP	CLC	Both	2000	Retrospective	Imura et al. (2007)^30^
12 Months/100%	GP/CaOH	WVC	Primary	50	Retrospective	Cotton et al. (2008)^31^
12 Months/100%	MTA	AP	Primary	22	Prospective	Moore et al. (2011)^32^
12 Months/100%	MTA + CaOH	AP	Primary	30	Randomized	Damle et al. (2012)^33^
12 Months/81%	GP/AH Plus	WVC	Primary	151	Prospective	Patel et al. (2012)^34^
12 Months/100%	GP/ZOE	CLC	Primary	167	Randomized	Saini et al. (2012)^35^
12 Months/88%	MTA	AP	Both	287	Retrospective	Mente et al. (2013)^36^
12 Months/80%	GP/AH Plus	WVC	Primary	105	Randomized	Liang et al. (2013)^37^
12 Months/100%	GP/AH Plus	CLC	Primary	360	Randomized	Tang et al. (2015)^38^
12 Months/86%	GP/Sealer not mentioned	CB	Primary	256	Randomized	Wong et al. (2015)^39^
12 Months/93%	GP/AH Plus	CLC	Primary	28	Randomized	Claudia et al. (2016)^40^
12 Months/100%	GP/CaOH	CLC	Primary	140	Retrospective	Sarin et al. (2016)^41^
12 Months/84%	GP/AH Plus	WVC	Primary	89	Prospective	Sigurdson et al. (2016)^42^
12 Months/100%	MTA	AP	Primary	21	Retrospective	Chen et al. (2016)^43^
12 Months/77%	GP/Sealer not mentioned	CLC	Primary	60	Prospective	Arya et al. (2017)^44^
12 Months/89%	CaOH	AP	Primary	38	Randomized	Lin et al. (2017)^45^
12 Months/87%	GP/CaOH	WVC	Retreatment	156	Prospective	Al-nuaimi et al. (2017)^46^
12 Months/93%	GP/ZOE	CLC	Primary	27	Randomized	Galani et al. (2017)^47^
12 Months/98%	GP/AH Plus	WVC	Primary	45	Prospective	Sigurdsson et al. (2018)^48^
12 Months/100%	GP/BC	SC	Both	307	Retrospective	Elizabeth et al. (2018)^49^
12 Months/86%	GP/ZOE	CLC	Primary	100	Randomized	Verma et al. (2019)^50^
12 Months/84%	GP/Resin Based	CLC	Primary	50	Randomized	Arslan et al. (2019)^51^
12 months/92%	GP/ZOE	CLC	Primary	120	Randomized	Kumar et al. (2020)^52^
12 Months/90%	GP/ZOE	WVC	Primary	242	Retrospective	Best et al. (2021)^53^
12 Months/86%	GP/AH Plus	CLC	Retreatment	214	Retrospective	Conejero et al. (2021)^54^
12 Months/100%	GP/AH Plus	TH	Primary	60	Prospective	Koli et al. (2021)^55^
12 Months/82%	GP/BC + ZOE	WVC SC	Both	84	Randomized	Bardini et al. (2021)^56^
12 Months/96%	GP/ZOE	CLC	Primary	120	Randomized	Fatima et al. (2021)^57^
12 Months/100%	GP/Resin Based	CLC	Retreatment	30	Retrospective	Tosun et al. (2021)^58^
12 Months/80%	GP/AH Plus	WVC	Primary	144	Randomized	Zahran et al. (2021)^59^
12 Months/86%	GP/AH Plus	SC	Secondary	120	Prospective	Serefoglu et al. (2021)^60^
12 Months/86%	GP/Resin based	SC	Primary	14	Randomized	Ahmed et al. (2023)^61^
12 Months/97%	GP/Sealer not mentioned	CLC	Primary	30	Randomized	Taha et al. (2023)^62^
12 Months/85%	GP/AH Plus + BC	WVC SC	Primary	181	Randomized	Alzoubi et al. (2025)^63^
12 Months/100%	GP/Resin Based+ BC	WVC SC	Primary	238	Retrospective	Bani-Younes et al. (2025)^64^
12 Months/100%	GP/Resin Based	CLC	Primary	70	Randomized	Doğan et al. (2024)^65^
24 Months/100%	GP/ZOE	WVC	Primary	223	Retrospective	Field et al. (2004)^66^
24 Months/94%	GP/ZOE	WVC	Retreatment	451	Retrospective	Gorni et al. (2004)^67^
24 Months/100%	GP/Resin Based	CLC	Retreatment	75	Retrospective	Caliskan et al. (2005)^68^
24 Months/88%	GP/Resin Based	CLC	Primary	101	Randomized	Molander et al. (2007)^69^
24 Months/100%	MTA	AP	Both	20	Retrospective	Holden et al. (2008)^70^
24 Months/100%	CaOH	AP	Primary	28	Prospective	Mendoza et al. (2010)^71^
24 months/100%	GP/Sealer not mentioned	CLC	Primary	105	Randomized	Tootla et al. (2012)^72^
24 Months/94%	GP/CaOH	CLC	Primary	300	Randomized	Vieyra et al. (2012)^73^
24 Months/75%	GP/ZOE	SC	Primary	71	Randomized	Jordan et al. (2014)^74^
24 Months/93%	GP/AH Plus	CLC CB	Primary	120	Randomized	Demirci et al. (2016)^75^
24 Months/100%	GP/CaOH	WVC	Retreatment	49	Prospective	Nešković et al. (2016)^76^
24 Months/89%	GP/ZOE MTA	WVC AP	Both	36	Randomized	Alsulaimani et al. (2016)^77^
24 Months/91%	GP/AH Plus	CLC	Primary	90	Prospective	Kurt et al. (2018)^78^
24 Months/92%	GP/Resin based	SC	Primary	75	Randomized	Cunha et al. (2020)^79^
24 Months/100%	GP/AH Plus	WVC	Primary	176	Prospective	Gudac et al. (2021)^80^
24 Months/96%	GP/Resin Based	CLC	Primary	51	Randomized	Asgary et al. (2022)^81^
24 Months/89%	GP/AH Plus	CLC	Retreatment	100	Randomized	Karaoğlan et al. (2022)^82^
24 Months/82%	GP/CaOH	SC	Primary	318	Retrospective	Subbiya et al. (2022)^83^
24 Months/88%	GP/AH Plus + BC	CLC	Primary	100	Randomized	Coşar et al. (2023)^84^
24 Months/97%	GP/AH Plus + BC	CB	Both	92	Prospective	Zamparini et al. (2023)^85^
24 Months/100%	GP/ZOE	WVC	Retreatment	120	Retrospective	Baltieri et al. (2024)^86^
24 Months/90%	GP/AH Plus	WVC	Primary	117	Randomized	Cagidiaco et al. (2025)^87^
24 Months/86%	GP/BC	SC	Both	140	Randomized	Simnon et al. (2025)^88^
3 + Years/94%	GP/Sealer not mentioned	CLC	Retreatment	54	Prospective	Sundqvist et al. (1998)^89^
3 + Years/92%	GP/CaOH	CLC	Primary	73	Prospective	Weiger et al. (2000)^90^
3 + Years/100%	GP/Resin based	WLC	Primary	39	Prospective	Peters et al. (2002)^91^
3 + Years/84%	GP/AH Plus	CLC	Primary	85	Prospective	Chu et al. (2005)^82^
3 + Years/100%	GP/AH Plus	CLC	Primary	340	Prospective	Aqrabawy et al. (2006)^93^
3 + Years/82%	GP/Sealer not mentioned	CLC CB	Primary	98	Randomized	Ozer et al. (2009)^94^
3 + Years/87%	GP/ZOE	CLC	Primary	60	Prospective	Riccitiello et al. (2011)^95^
3 + Years/90%	GP/AH Plus	CB	Both	213	Retrospective	Pirani et al. (2016)^96^
3 + Years/100%	GP/AH Plus	CB	Both	206	Retrospective	Chiara et al. (2019)^97^
3 + Years/87%	GP/AH Plus	CLC	Retreatment	52	Randomized	Zandi et al. (2019)^98^
3 + years/86%	GP/AH Plus	CLC	Primary	100	Randomized	Kurt et al. (2022)^99^
3 + Years/77%	GP/ZOE + BC	WVC SC	Both	67	Randomized	Bardini et al. (2024)^100^

***Abbreviations***: CLC, cold lateral compaction; CB, carrier-based technique; SC, single cone, Randomized, randomized controlled trial; GP, gutta-percha; ZOE, zinc oxide eugenol; BC, bioceramic; MTA, mineral trioxide aggregate; TH, thermoplastiscized; AP, apexification.

### Characteristics of included studies

The analysis incorporated 11,965 samples from 84 studies, consisting of 42 randomized controlled trials and 42 cohort studies (prospective and retrospective). These studies focused on RCT, re-RT, and apexification, categorized by different follow-up periods.

For primary root canal treatment, studies were grouped by follow-up intervals. Nine studies evaluated treatments over a 6-month period, analyzing four techniques: WVC, SC, CLC, and CB. For the 12-month period, 32 studies investigated six techniques, including WVC, SC, CLC, TH, and CB. Eighteen studies reported results for the 24-month period, covering WVC, SC, CLC, and CB techniques. Ten studies assessed outcomes at three or more years, with comparisons between WVC, WLC, CLC, SC, and CB techniques.

For retreatment procedures, two studies reported results at 6 months, evaluating SC and CLC techniques. At 12 months, ten studies examined WVC, SC, and CLC, while eight studies analyzed WVC, CLC, SC, and CB at 24 months. Results for follow-up beyond three years were reported in five studies, focusing on CLC, WVC, SC, and CB techniques. Additionally, nine articles explored the apexification technique across various follow-up durations.

### Meta-analysis

**Fig. 2:**
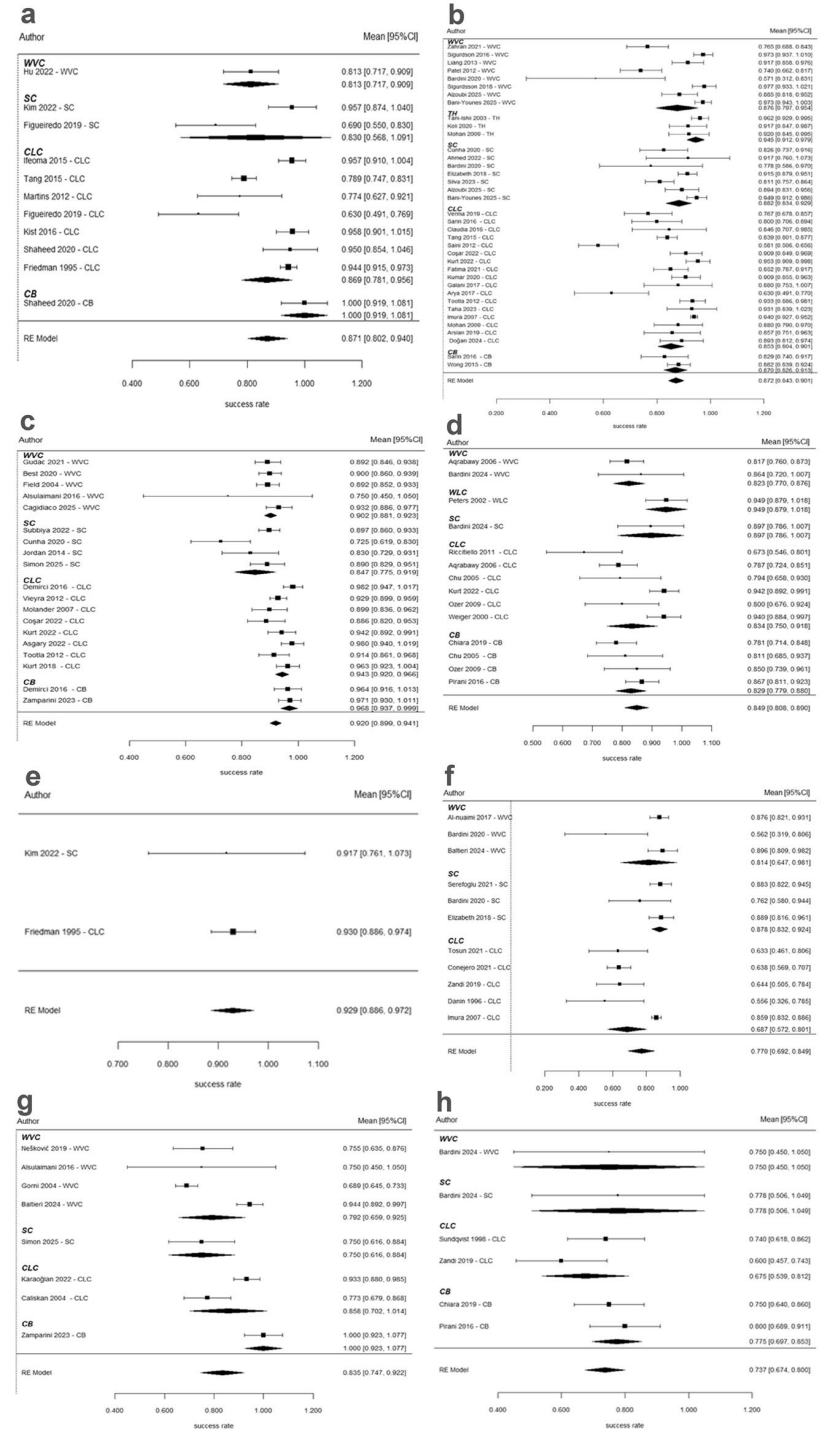
Forest plots for primary root canal treatments at different follow-up periods: (a) 6 months, (b) 12 months, (c) 24 months, and (d) 3 + years. For root canal retreatments, the outcomes are presented for (e) 6 months, (f) 12 months, (g) 24 months, and (h) 3 + years.

The success rate for root canal filling techniques was estimated using meta-analysis, with follow-up durations serving as the moderator variable is illustrated in Fig. 2. For primary root canal treatment, the overall success rate at six months was 87.1% (95% CI: 80.2–94.0). No statistically significant differences were observed between techniques; for instance, SC showed a non-significant 3.2% lower success rate compared to CLC (*p* = 0.766). At 12 months, the success rate remained 87.2% (95%CI: 84.3–90.1), with no significant differences across techniques (*p* = 0.677). By 24 months, the success rate improved to 92.0% (95%CI: 89.9–94.1), with CLC and CB techniques demonstrating significantly better results compared to WVC (*p* = 0.021 and *p* = 0.011, respectively), Furthermore, SC exhibited significantly worse results compared to both CLC and CB (*p* = 0.001). The success rate beyond three years was 84.9% (95%CI: 80.8–89.0), and no significant differences between techniques were observed.

For retreatment procedures, the overall success rate at six months was 92.9% (95% CI: 88.6–97.2); however, limited data restricted further comparisons. At 12 months, the success rate was 77.0% (95%CI: 69.2–84.9), with SC demonstrating a marginally significant improvement of 16.7% over CLC (*p* = 0.045). The success rate at 24 months was 83.5% (95%CI: 74.7–92.2), with CB performing significantly better than WVC (*p* = 0.004). Beyond three years, the overall success rate dropped to 73.7% (95%CI: 67.4–80.0); although CB showed a 7.9% improvement over CLC, the difference was not statistically significant (*p* = 0.179). A separate analysis revealed that the apexification technique demonstrated significantly higher success rates compared to CLC at the 12-month follow-up (*p* = 0.019) (Suppl. Figure 1).

### Meta-regression analysis

The meta-regression analysis examined the impact of various independent factors, including mean age, gender distribution, tooth position, operator expertise, and materials used (Suppl. Table 2). Tooth position significantly influenced outcomes, with maxillary teeth showing superior success rates (*p* = 0.006). Operator expertise also played a critical role, with specialists achieving significantly higher success rates compared to general dentists and students (*p* < 0.001). Material comparisons revealed marginal significance for zinc oxide eugenol compared to AH-Plus or glass ionomer cements (*p* = 0.091 and *p* = 0.080, respectively).

### Qualitative assessment

**Fig. 3:**
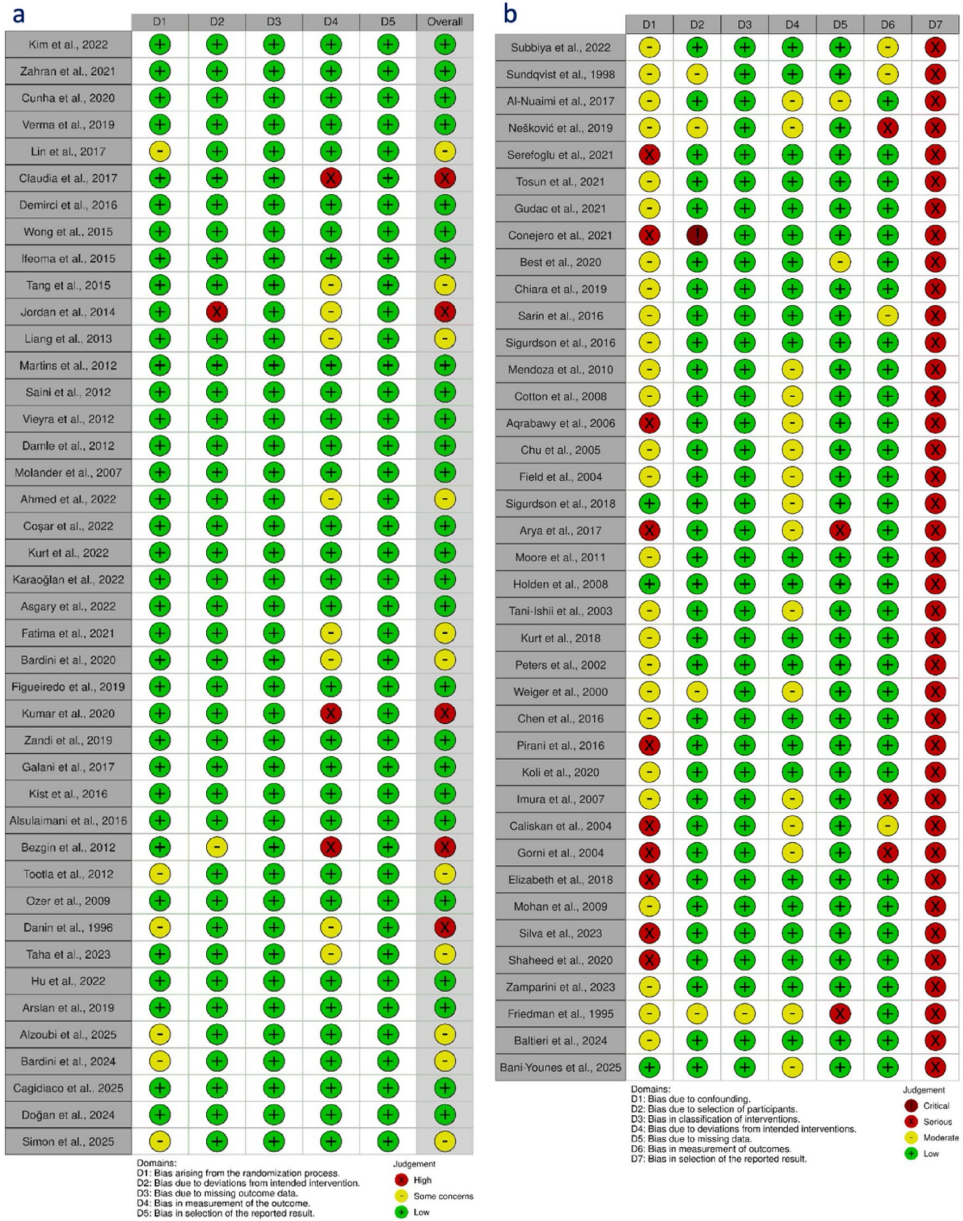
Summary of risk of bias assessment using (a) ROB 2.0 for randomized clinical trials and (b) ROBINS-I for non-randomized studies.

Risk of bias was assessed using RoB 2.0 (randomized) and ROBINS-I (cohorts). Among RCTs, 27 had “some concerns,” others were low risk. Cohort studies mostly had serious bias, especially in outcome reporting; one study had critical participant selection bias (Fig. 3). GRADE assessment rated five outcomes as very low and three as low certainty, including 12- and 24-month RCT outcomes and 12-month re-RCT results (Table 3).

**Table 3:** Summary of findings using the GRADE approach for evaluating clinical and radiographic outcomes.

Outcomes	Anticipated absolute effects (95% CI)	Relative effect (95% CI)	Number of teeth (studies, *n*)	Certainty of the evidence (GRADE)	Comments
RCT success at 6 months	871 (802 to 940)	0.871 (0.802, 0.940)	974 (9)	⊕◯◯◯ Very low	The evidence for the effect of root canal treatment at 6 months is very uncertain due to the very low certainty of evidence. This is attributed to limitations in study design, relatively small sample size, and potential variability across studies.
RCT success at 12 months	862 (830 to 895)	0.862 (0.830, 0.895)	4792 (32)	⊕⊕◯◯ Low	The results suggest that root canal treatment at 12 months has a stable outcome, supported by a large sample size and narrow confidence intervals. However, the evidence is downgraded to low certainty due to potential risk of bias or heterogeneity among the included studies.
RCT success at 24 months	920 (896 to 943)	0.920 (0.896, 0.943)	2187 (18)	⊕⊕◯◯ Low	The findings at 24 months indicate consistent treatment success, with narrow confidence intervals and a moderate sample size. Despite these strengths, the certainty of evidence is low, likely due to concerns about study design and the variability in study methodologies.
RCT success at 3 + years	844 (798 to 890)	0.844 (0.798, 0.890)	1093 (10)	⊕◯◯◯ Very low	The evidence for RCT outcomes at 3 + years is very uncertain due to very low certainty of evidence. This is caused by a relatively small sample size, broader confidence intervals, and potential issues with study quality and variability.
Re-RCT success at 6 months	929 (886 to 972)	0.929 (0.886, 0.972)	140 (2)	⊕◯◯◯ Very low	The results for re-RCT outcomes at 6 months are very uncertain. The small sample size and potential heterogeneity among studies significantly limit the reliability of the evidence, leading to a very low certainty rating.
Re-RCT success at 12 months	756 (672 to 839)	0.756 (0.672, 0.839)	1299 (10)	⊕⊕◯◯ Low	Re-RCT outcomes at 12 months indicate modest effectiveness, with a relatively narrow confidence interval and a larger sample size. However, the certainty of evidence remains low due to concerns about study design and consistency among included studies.
Re-RCT success at 24 months	825 (717 to 933)	0.825 (0.717, 0.933)	779 (8)	⊕◯◯◯ Very low	The evidence for re-RCT outcomes at 24 months is very uncertain due to the very low certainty rating. This is attributed to the smaller sample size, wide confidence intervals, and study design limitations.
Re-RCT success at 3 + years	732 (659 to 806)	0.732 (0.659, 0.806)	222 (5)	⊕◯◯◯ Very low	The evidence for re-RCT outcomes at 3 + years is very uncertain due to very low certainty of evidence. The small sample size and broad confidence intervals suggest a lack of precision in the results.

***Abbreviations***: RCT, root canal treatment; Re-RCT retreatment case.

## Discussion

This systematic review and meta-analysis evaluated the clinical and radiographic success rates of root canal filling techniques and materials in both primary RCTs and re-RCT across different follow-up periods. The study incorporated an extensive search strategy and included both randomized controlled trials and cohort studies to ensure robust and reliable findings.

*Primary root canal treatments*.

The results of the meta-analyses for primary RCTs showed consistently high overall success rates at the 6- and 12-month follow-up periods, with no statistically significant differences observed between techniques such as SC, CLC, WVC, and CB. These findings align with previous systematic reviews^101,102^, which similarly reported comparable outcomes among various techniques in short-term follow-ups. The SC technique, in particular, has gained clinical popularity due to its simplicity and the growing use of bioceramic sealers, which are desirable for their bioactivity and sealing properties^103,104^.

Within the limitations of current evidence, significant differences emerged at the 24-month follow-up period, where SC exhibited lower success rates compared to CLC and CB techniques, which showed 8.8% and 11.3% higher success, respectively. These results may reflect the better adaptation of filling materials achieved with CLC and CB, particularly in more challenging long-term scenarios. However, these differences could also be attributed to factors beyond the inherent sealing ability of the techniques. Variations in sample size and study design can significantly influence statistical outcomes, potentially exaggerating the effectiveness of one technique over another. For instance, variations in sample size and study design can introduce statistical bias, potentially exaggerating the effectiveness of one technique over another. Therefore, further high-quality randomized controlled trials are needed to confirm clinical significance.

A systematic review reported an overall success rate of 83% for core-carrier obturation but noted considerable variability among included studies, demonstrating the impact of sample size and methodology on outcomes^105^. Additionally, differences in case selection, such as tooth type, canal anatomy, and preoperative conditions, may have contributed to the observed success rates. Studies have shown that teeth with simpler anatomies or fewer complications are more likely to yield favorable outcomes, irrespective of the obturation technique used^11^.

Operator expertise includes not only technical proficiency but also case selection, complication management, and adherence to aseptic protocols. Moreover, clinicians’ familiarity with specific obturation methods can directly affect the quality of the root canal filling and treatment outcomes. These facets together are fundamental in achieving successful endodontic therapy. For instance, a study comparing CB and lateral compaction techniques concluded that both methods could achieve comparable success rates, with outcomes heavily influenced by operator skill rather than the technique itself^106^. These findings suggest that the apparent success observed with CLC and CB techniques at 24 months is likely multifactorial, involving variables such as case complexity, operator expertise, and study design rather than solely reflecting the sealing capabilities of the obturation materials. Despite these findings, success rates for all techniques converged beyond the 3-year follow-up period, with no statistically significant differences reported among WVC, WLC, CLC, and CB, suggesting that long-term clinical outcomes may be determined by factors unrelated to the technique itself. However, these differences were not sustained beyond 3 years, where outcomes converged across techniques. Given the low/very-low certainty of evidence and the influence of operator expertise and case selection, these intermediate differences should not be interpreted as technique-dependent superiority.

### Root Canal retreatment outcomes

Retreatment analyses were limited by the scarcity of available studies, particularly for the 6-month follow-up period, where only two studies were included^17,25^. At the 12-month interval, analyses incorporating WVC, CLC, and SC techniques revealed no significant differences in success rates. Similarly, the 24-month follow-up data demonstrated comparable outcomes between WVC, CLC, SC, and CB techniques. Interestingly, CB showed a numerical advantage over CLC beyond the 3-year follow-up, though statistical significance was not achieved. These findings accentuate the need for additional high-quality studies investigating retreatment procedures to draw more reliable conclusions.

### Influence of bioceramic sealers

The SC technique has been closely associated with bioceramic sealers, which have emerged as a promising alternative to traditional epoxy resin-based cements (e.g., AH-Plus). While bioceramic sealers have shown excellent biocompatibility, biomineralization potential, and enhanced sealing properties^96–99^, their long-term clinical outcomes remain inconclusive. Consistent with our findings, no long-term clinical advantage of bioceramic sealers over AH Plus has been demonstrated to date^102^. Most supportive evidence derives from in vitro or short-term studies, and the difficulties in retreatment removal remain unresolved. These findings are consistent with prior reviews^107^, which highlight the need for long-term, well-designed clinical trials to validate the clinical benefits of bioceramic materials.

Despite their advantages, bioceramic sealers pose challenges in retreatment scenarios due to the difficulty in removing them efficiently from the root canal system^108^. This limitation denotes the importance of careful case selection and the development of standardized protocols for their removal, particularly in cases requiring further intervention.

### Clinical implications

While newer obturation techniques and materials may offer certain practical advantages, no single approach consistently outperforms others across follow-up periods. Modest 24-month (mid-term) differences observed for some techniques (slightly higher success of CLB and CB) should be interpreted with caution^109^, as operator expertise and case selection are likely more decisive factors. Long-term success remains multifactorial and is less dependent on technique, suggesting that operator expertise, case complexity, and patient-specific factors play a central role in treatment success. Nonetheless, given that many outcomes were rated as having low or very low certainty of evidence, these findings should be interpreted with caution, and strong clinical recommendations cannot be made at this stage.

Operator experience emerged as a critical factor influencing success rates, with specialists achieving significantly better outcomes compared to general practitioners and students. This finding supports advanced training and skill development in endodontics to optimize clinical outcomes. Additionally, anatomical factors, such as differences between maxillary and mandibular teeth, were associated with varying success rates, with maxillary teeth exhibiting higher success, possibly due to their more accessible anatomy.

### Methodological amendments

This study incorporated key deviations from the initial protocol to improve clinical relevance. For instance, the inclusion criteria were adjusted to allow a dropout rate of up to 25%, as attrition rates above 20–30% are known to increase the risk of bias and compromise the validity of study outcomes. This threshold was selected to maintain methodological rigor while enabling the inclusion of a broader range of relevant endodontic clinical studies, consistent with approaches used in previous systematic reviews. Data extraction and subgroup analyses included additional factors such as tooth position, operator expertise, and material adaptation, enabling a more comprehensive assessment of treatment outcomes. To address heterogeneity, a mixed-effects meta-regression model replaced separate network meta-analyses, providing a standardized approach to outcome assessment across diverse time intervals. Quality assessment integrated ROBINS-I and the GRADE system alongside RoB 2, permitting a robust evaluation of bias and evidence certainty. Additionally, special attention was given to the impact of bioceramic sealers, particularly their influence on retreatment challenges and long-term clinical success.

### Strengths and limitations

The strengths of this review include a comprehensive search strategy, adherence to PRISMA guidelines, and rigorous quality assessment using the ROB 2.0, ROBINS-I, and GRADE tools. This systematic review and meta-analysis included a wide range of studies, offering valuable insights into the performance of various obturation techniques and sealers. However, the inclusion of both RCTs and cohort studies, variation in operator expertise and case selection, and differences in follow-up durations (ranging from 6 months to over 3 years) limit direct comparability of results and contribute to heterogeneity. These factors underscore the need for cautious interpretation of pooled estimates. Nevertheless, certain limitations must be acknowledged. Given the considerable methodological heterogeneity among included studies, including the limited number of studies assessing retreatment procedures, particularly with shorter follow-up periods, differences in operator training, case selection, and follow-up intervals, our conclusions must be interpreted with caution. Although trends were identified, the low certainty of evidence as per GRADE and the limited number of studies available (particularly in re-RCTs cases), limit the formulation of strong clinical recommendations based solely on the study findings. Publication bias, though assessed, cannot be entirely ruled out. Consequently, no conclusive clinical practice guidelines can be proposed at this stage.

### Further research

Future studies should prioritize well-designed, randomized controlled trials with standardized protocols for evaluating root canal filling techniques and materials to confirm clinical significance. Long-term follow-up data are essential to better understand the success and clinical impact of bioceramic sealers and other emerging materials. Additionally, further research is needed to develop efficient retreatment protocols for bioceramic-based fillings to address their limitations in re-intervention scenarios. Incorporating patient-reported outcomes (PROs) and tooth survival rates could provide a more comprehensive understanding of the effectiveness of RCTs and re-RCTs.

## Conclusion

Within the limits of this secondary study, root canal filling techniques and materials demonstrated comparable clinical and radiographic success in short-term follow-ups. Some intermediate-term differences were observed, but outcomes converged beyond 3 years. Operator expertise, case selection, and patient-specific factors appear more influential than the obturation technique itself. Bioceramic sealers have not consistently demonstrated long-term clinical advantage over AH Plus, with most evidence limited to in vitro or short-term studies, and challenges in retreatment remain. Given the low to very low certainty of evidence, these findings should be interpreted with caution, understanding the need for well-designed long-term randomized controlled trials with standardized protocols to strengthen evidence-based guidance in endodontic therapy.

## Supplementary Information

Below is the link to the electronic supplementary material.

Supplementary Material 1

## Data Availability

The datasets generated during and/or analyzed during the current study are available from the corresponding author on reasonable request.
