# Supplementary material for: Effect of root canal filling techniques and materials on endodontic treatment outcomes: a systematic review and meta-analysis
Source: Sci Rep. 2026 Mar 23;16:9552. doi: 10.1038/s41598-026-37936-7 (PMC13009500; doi:10.1038/s41598-026-37936-7)
Supplement: Supplementary file 1 — Supplementary Material 1 [file 41598_2026_37936_MOESM1_ESM.docx]

**SUPPLEMENTARY MATERIAL**

| **Study ID** | **Sample Size** | **Obturation Technique Mentioned** | **Follow-Up Period** | **Drop-Out Rate** | **Radiographic Assessment** | **Signs and Symptoms** |
| --- | --- | --- | --- | --- | --- | --- |
| **Wang et al., (2023)** | **✔** | **X** | **X** | **X** | **✔** | **✔** |
| **Stueland et al., (2023)** | **✔** | **X** | **✔** | **X** | **✔** | **X** |
| **Van Nieuwenhuysen et al., (2023)** | **✔** | **X** | **✔** | **X** | **✔** | **✔** |
| **Johnsen et al., (2023)** | **✔** | **X** | **✔** | **X** | **✔** | **✔** |
| **Paljevic et al., (2023)** | **✔** | **X** | **✔** | **✔** | **✔** | **✔** |
| **Peña-Bengoa et al., (2023)** | **✔** | **X** | **X** | **X** | **✔** | **X** |
| **Almeida et al., (2023)** | **✔** | **X** | **✔** | **X** | **✔** | **✔** |
| **Martins et al., (2023)** | **✔** | **X** | **✔** | **✔** | **✔** | **✔** |
| **Saini et al., (2023)** | **✔** | **X** | **✔** | **✔** | **✔** | **✔** |
| **Makanjuola et al., (2023)** | **✔** | **X** | **✔** | **X** | **✔** | **✔** |
| **Yanni et al., (2022)** | **✔** | **X** | **✔** | **X** | **X** | **X** |
| **Lee et al., (2022)** | **✔** | **X** | **✔** | **✔** | **✔** | **X** |
| **Burns et al., (2022)** | **✔** | **X** | **✔** | **X** | **X** | **X** |
| **Guo et al., (2022)** | **✔** | **X** | **✔** | **✔** | **✔** | **✔** |
| **Lu et al., (2022)** | **✔** | **✔** | **✔** | **✔** | **X** | **X** |
| **Ferracane et al., (2022)** | **✔** | **X** | **✔** | **✔** | **X** | **✔** |
| **Guang et al., (2022)** | **✔** | **X** | **✔** | **✔** | **✔** | **✔** |
| **Herbst et al., (2022)** | **✔** | **X** | **✔** | **X** | **✔** | **✔** |
| **Shahmohammadi et al., (2021)** | **✔** | **X** | **✔** | **✔** | **✔** | **✔** |
| **Bhagavatula et al., (2021)** | **✔** | **X** | **✔** | **X** | **X** | **X** |
| **Haxhia et al., (2021)** | **✔** | **X** | **✔** | **X** | **X** | **X** |
| **Kosanwat et al., (2021)** | **✔** | **X** | **✔** | **X** | **X** | **X** |
| **Lee et al., (2021)** | **✔** | **X** | **✔** | **✔** | **X** | **X** |
| **Ptak et al., (2021)** | **✔** | **✔** | **✔** | **X** | **✔** | **X** |
| **Ruetters et al., (2021)** | **✔** | **X** | **✔** | **✔** | **X** | **X** |
| **Salah et al., (2021)** | **✔** | **✔** | **✔** | **X** | **✔** | **✔** |
| **Chen et al., (2021)** | **✔** | **X** | **✔** | **✔** | **X** | **X** |
| **Kebke et al., (2021)** | **✔** | **X** | **✔** | **✔** | **✔** | **✔** |
| **Laukkanen et al., (2021)** | **✔** | **X** | **✔** | **✔** | **✔** | **X** |
| **Wu et al., (2021)** | **✔** | **X** | **✔** | **✔** | **X** | **X** |
| **Al-Haddad et al., (2020)** | **✔** | **X** | **✔** | **✔** | **X** | **X** |
| **Zhang et al., (2020)** | **✔** | **X** | **✔** | **✔** | **X** | **X** |
| **García-Guerrero et al., (2020)** | **✔** | **X** | **✔** | **X** | **✔** | **✔** |
| **Eyüboğlu et al., (2020)** | **✔** | **✔** | **✔** | **X** | **✔** | **✔** |
| **Hinz et al., (2020)** | **✔** | **✔** | **✔** | **X** | **X** | **X** |
| **Brignardello., (2020)** | **✔** | **X** | **✔** | **X** | **X** | **X** |
| **Stenhagen et al., (2020)** | **✔** | **✔** | **✔** | **✔** | **✔** | **X** |
| **Fan et al., (2020)** | **✔** | **X** | **✔** | **✔** | **X** | **X** |
| **Alharmoodi et al., (2020)** | **✔** | **X** | **✔** | **✔** | **✔** | **X** |
| **Orozco et al., (2020)** | **✔** | **X** | **✔** | **X** | **✔** | **✔** |
| **Goldberg et al., (2020)** | **✔** | **✔** | **✔** | **X** | **✔** | **X** |
| **Mahmood et al., (2019)** | **✔** | **X** | **X** | **X** | **✔** | **X** |
| **Restrepo-Restrepo et al., (2019)** | **✔** | **✔** | **✔** | **✔** | **✔** | **✔** |
| **Laukkanen et al., (2019)** | **✔** | **✔** | **✔** | **✔** | **✔** | **X** |
| **Kwak et al., (2019)** | **✔** | **X** | **✔** | **✔** | **✔** | **✔** |
| **Laukkanen et al., (2019)** | **✔** | **✔** | **✔** | **✔** | **✔** | **X** |
| **Olcay et al., (2019)** | **✔** | **✔** | **✔** | **X** | **✔** | **✔** |
| **Jahreis et al., (2019)** | **✔** | **✔** | **✔** | **X** | **✔** | **✔** |
| **Sun et al., (2019)** | **✔** | **X** | **✔** | **X** | **X** | **X** |
| **Fezai et al., (2019)** | **✔** | **X** | **X** | **X** | **✔** | **X** |
| **Barbosa-Ribeiro et al., (2019)** | **✔** | **✔** | **✔** | **✔** | **X** | **✔** |
| **Alves de Melo et al., (2019)** | **✔** | **X** | **✔** | **✔** | **X** | **X** |
| **Vahdati et al., (2019)** | **✔** | **X** | **✔** | **X** | **✔** | **✔** |
| **Chung et al., (2019)** | **✔** | **✔** | **✔** | **X** | **✔** | **✔** |
| **Machado et al., (2019)** | **✔** | **✔** | **X** | **X** | **X** | **✔** |
| **Lee et al., (2019)** | **X** | **✔** | **X** | **✔** | **✔** | **✔** |
| **Duque et al., (2019)** | **✔** | **X** | **✔** | **✔** | **X** | **X** |
| **Morsy et al., (2018)** | **✔** | **✔** | **✔** | **X** | **X** | **✔** |
| **Riis et al., (2018)** | **✔** | **✔** | **✔** | **✔** | **✔** | **✔** |
| **Dias et al., (2018)** | **✔** | **X** | **✔** | **✔** | **X** | **X** |
| **de Miranda et al., (2018)** | **✔** | **✔** | **✔** | **✔** | **✔** | **✔** |
| **Yamaguchi et al., (2018)** | **✔** | **X** | **X** | **X** | **X** | **X** |
| **Lin et al., (2018)** | **✔** | **X** | **✔** | **✔** | **X** | **X** |
| **Beus et al., (2018)** | **✔** | **X** | **✔** | **✔** | **X** | **X** |
| **Raslan et al., (2017)** | **✔** | **X** | **✔** | **✔** | **X** | **X** |
| **Monaco et al., (2017)** | **✔** | **X** | **✔** | **✔** | **✔** | **X** |
| **Agrafioti et al., (2017)** | **✔** | **X** | **X** | **X** | **X** | **X** |
| **Jorgensen et al., (2017)** | **✔** | **X** | **✔** | **✔** | **X** | **X** |
| **Eyuboglu et al., (2017)** | **✔** | **✔** | **✔** | **X** | **✔** | **✔** |
| **Dumani et al., (2017)** | **✔** | **X** | **✔** | **✔** | **X** | **X** |
| **Krug et al., (2017)** | **✔** | **✔** | **✔** | **X** | **✔** | **X** |
| **Chang et al., (2017)** | **✔** | **✔** | **✔** | **X** | **✔** | **✔** |
| **Cloet et al., (2017)** | **✔** | **X** | **✔** | **✔** | **✔** | **X** |
| **Priyank et al., (2016)** | **✔** | **X** | **X** | **X** | **X** | **✔** |
| **Tsarev et al., (2016)** | **✔** | **X** | **✔** | **✔** | **X** | **X** |
| **Asgary et al., (2016)** | **✔** | **X** | **✔** | **✔** | **X** | **X** |
| **Garcez et al., (2015)** | **✔** | **X** | **✔** | **✔** | **X** | **X** |
| **Zuolo et al., (2015)** | **✔** | **X** | **X** | **X** | **X** | **X** |
| **Colaco and Pai., (2015)** | **✔** | **X** | **✔** | **✔** | **X** | **X** |
| **Clarke et al., (2015)** | **✔** | **✔** | **✔** | **X** | **✔** | **X** |
| **Martinho et al., (2015)** | **✔** | **X** | **X** | **X** | **✔** | **✔** |
| **Davies et al., (2015)** | **✔** | **X** | **X** | **X** | **✔** | **X** |
| **Biezanek et al., (2015)** | **✔** | **X** | **X** | **✔** | **X** | **X** |
| **Jurič et al., (2014)** | **✔** | **✔** | **X** | **X** | **✔** | **X** |
| **Estrela et al., (2014)** | **✔** | **✔** | **✔** | **X** | **✔** | **X** |
| **Alobaid et al., (2014)** | **✔** | **✔** | **✔** | **X** | **✔** | **✔** |
| **Juloski et al., (2014)** | **✔** | **✔** | **✔** | **✔** | **✔** | **X** |
| **Martinho et al., (2014)** | **✔** | **X** | **X** | **X** | **X** | **✔** |
| **Kahler et al., (2014)** | **✔** | **X** | **✔** | **X** | **X** | **X** |
| **Song et al., (2014)** | **✔** | **X** | **X** | **X** | **✔** | **X** |
| **Fullmer et al., (2014)** | **✔** | **X** | **X** | **X** | **X** | **X** |
| **Krupp et al., (2013)** | **✔** | **X** | **X** | **X** | **✔** | **X** |
| **Jin et al., (2013)** | **✔** | **X** | **X** | **X** | **✔** | **X** |
| **van der Borden et al., (2013)** | **✔** | **✔** | **✔** | **X** | **✔** | **X** |
| **Xavier et al., (2013)** | **✔** | **X** | **X** | **X** | **X** | **✔** |
| **Akbar et al., (2013)** | **✔** | **✔** | **X** | **✔** | **X** | **✔** |
| **Vozza et al., (2013)** | **✔** | **X** | **✔** | **X** | **✔** | **X** |
| **Sterzenbach et al., (2012)** | **✔** | **X** | **✔** | **✔** | **✔** | **X** |
| **Liang et al., (2012)** | **✔** | **✔** | **✔** | **X** | **✔** | **✔** |
| **Pawar et al., (2012)** | **✔** | **X** | **X** | **✔** | **X** | **X** |
| **Oliveira et al., (2012)** | **✔** | **✔** | **✔** | **X** | **X** | **✔** |
| **Ferrari et al., (2012)** | **✔** | **X** | **✔** | **✔** | **✔** | **✔** |
| **Kangarlou et al., (2014)** | **✔** | **X** | **✔** | **✔** | **X** | **X** |
| **Munoz et al., (2012)** | **✔** | **X** | **✔** | **✔** | **X** | **X** |
| **Abramovitz et al., (2012)** | **✔** | **X** | **✔** | **✔** | **X** | **X** |
| **Dahlström et al., (2011)** | **✔** | **✔** | **✔** | **X** | **✔** | **X** |
| **Beslot-Neveu et al., (2011)** | **✔** | **X** | **✔** | **✔** | **X** | **X** |
| **Prashanth et al., (2011)** | **✔** | **✔** | **X** | **X** | **✔** | **✔** |
| **Signore et al., (2011)** | **✔** | **✔** | **✔** | **✔** | **X** | **✔** |
| **Simpson et al., (2011)** | **✔** | **X** | **✔** | **X** | **X** | **X** |
| **Liang et al., (2011)** | **✔** | **✔** | **✔** | **X** | **✔** | **✔** |
| **Marfisi et al., (2010)** | **✔** | **X** | **X** | **X** | **X** | **X** |
| **Gómez-Polo et al., (2010)** | **✔** | **X** | **✔** | **✔** | **✔** | **✔** |
| **Ahrari et al., (2010)** | **✔** | **X** | **✔** | **X** | **✔** | **X** |
| **Lee et al., (2010)** | **✔** | **✔** | **X** | **X** | **✔** | **✔** |
| **Fleming et al., (2010)** | **✔** | **✔** | **✔** | **✔** | **X** | **X** |
| **Gomes et al., (2009)** | **✔** | **X** | **X** | **X** | **X** | **✔** |
| **Taschieri et al., (2009)** | **✔** | **X** | **✔** | **X** | **X** | **✔** |
| **Mannocci et al., (2002)** | **✔** | **✔** | **✔** | **X** | **✔** | **✔** |
| **Signore et al., (2009)** | **✔** | **✔** | **✔** | **✔** | **X** | **X** |
| **Hannahan et al., (2008)** | **✔** | **X** | **✔** | **X** | **✔** | **✔** |
| **Risso et al., (2008)** | **✔** | **✔** | **X** | **X** | **X** | **✔** |
| **Taschieri et al., (2008)** | **✔** | **X** | **✔** | **✔** | **X** | **X** |
| **Só et al., (2008)** | **✔** | **X** | **X** | **X** | **X** | **X** |
| **Cagidiaco et al., (2008)** | **✔** | **✔** | **✔** | **✔** | **X** | **X** |
| **Penesis et al., (2008)** | **✔** | **✔** | **✔** | **X** | **✔** | **✔** |
| **Adolphi et al., (2007)** | **✔** | **X** | **✔** | **✔** | **X** | **X** |
| **Yazdi et al., (2007)** | **✔** | **X** | **✔** | **✔** | **✔** | **✔** |
| **Silveira et al., (2007)** | **✔** | **X** | **✔** | **✔** | **X** | **X** |
| **Ng et al., (2006)** | **✔** | **X** | **✔** | **✔** | **X** | **X** |
| **Kaufman et al., (2005)** | **✔** | **X** | **X** | **X** | **X** | **✔** |
| **Iqbal et al., (2003)** | **✔** | **X** | **X** | **X** | **✔** | **✔** |
| **Frajlich et al., (1998)** | **✔** | **✔** | **X** | **✔** | **✔** | **X** |
| **Özdemir et al., (2022)** | **✔** | **X** | **X** | **X** | **X** | **X** |
| **Jordal et al., (2022)** | **✔** | **X** | **✔** | **X** | **✔** | **X** |
| **Patel et al., (2022)** | **✔** | **X** | **✔** | **✔** | **✔** | **✔** |
| **Martinho et al., (2021)** | **✔** | **✔** | **✔** | **✔** | **X** | **X** |
| **Signor et al., (2021)** | **✔** | **X** | **✔** | **X** | **✔** | **✔** |
| **Karan et al., (2020)** | **✔** | **X** | **✔** | **✔** | **X** | **X** |
| **Buchgreitz et al., (2019)** | **✔** | **X** | **✔** | **✔** | **X** | **X** |
| **Dagher et al., (2019)** | **✔** | **✔** | **X** | **X** | **X** | **✔** |
| **Oliveira et al., (2019)** | **✔** | **X** | **X** | **X** | **X** | **✔** |
| **Riaz et al., (2018)** | **✔** | **✔** | **X** | **X** | **X** | **X** |
| **Nino-Barrera et al., (2018)** | **✔** | **✔** | **X** | **X** | **✔** | **✔** |
| **Dawson et al., (2017)** | **✔** | **X** | **✔** | **✔** | **X** | **X** |
| **Granevik Lindström et al., (2017)** | **✔** | **X** | **X** | **X** | **✔** | **X** |
| **Fonzar et al., (2017)** | **✔** | **✔** | **✔** | **✔** | **X** | **✔** |
| **Gupta et al., (2015)** | **✔** | **X** | **✔** | **✔** | **X** | **X** |
| **Lee et al., (2015)** | **✔** | **✔** | **X** | **X** | **✔** | **✔** |
| **Prati et al., (2014)** | **✔** | **X** | **✔** | **X** | **✔** | **X** |
| **Nagata et al., (2014)** | **✔** | **X** | **✔** | **✔** | **X** | **X** |
| **Inchingolo et al., (2014)** | **✔** | **X** | **✔** | **✔** | **X** | **X** |
| **Martins et al., (2014)** | **✔** | **✔** | **✔** | **X** | **✔** | **✔** |
| **Neelakantan et al., (2013)** | **✔** | **X** | **X** | **X** | **✔** | **X** |
| **Jadhav et al., (2012)** | **✔** | **X** | **✔** | **✔** | **X** | **X** |
| **Alsalleeh et al., (2024)** | **✔** | **✔** | **✔** | **X** | **✔** | **X** |
| **Jeeruphan et al., (2012)** | **✔** | **✔** | **✔** | **✔** | **X** | **X** |
| **Jeger et al., (2012)** | **✔** | **X** | **X** | **X** | **X** | **X** |
| **Jarad et al., (2011)** | **✔** | **✔** | **X** | **X** | **✔** | **X** |
| **Hsiao et al., (2009)** | **✔** | **X** | **✔** | **✔** | **X** | **X** |
| **Estrela et al., (2009)** | **✔** | **X** | **✔** | **✔** | **X** | **X** |
| **de Chevigny et al., (2008)** | **✔** | **X** | **✔** | **X** | **✔** | **✔** |
| **Simon et al., (2007)** | **✔** | **✔** | **✔** | **X** | **✔** | **X** |
| **Doyle et al., (2006)** | **✔** | **✔** | **✔** | **X** | **✔** | **✔** |
| **Tsesis et al., (2006)** | **✔** | **X** | **✔** | **✔** | **X** | **X** |
| **Chu et al., (2006)** | **✔** | **X** | **✔** | **X** | **X** | **✔** |
| **Waltimo et al., (2005)** | **✔** | **X** | **✔** | **X** | **✔** | **X** |
| **Quesnell et al., (2005)** | **✔** | **X** | **✔** | **✔** | **✔** | **X** |
| **Peters et al., (2004)** | **✔** | **✔** | **✔** | **✔** | **✔** | **X** |
| **Fristad et al., (2004)** | **✔** | **X** | **✔** | **✔** | **X** | **X** |
| **Mannocci et al., (2002)** | **✔** | **X** | **✔** | **X** | **✔** | **X** |
| **Kvist et al., (1999)** | **✔** | **X** | **✔** | **X** | **✔** | **✔** |
| **Eleazer et al., (1998)** | **✔** | **✔** | **X** | **X** | **X** | **✔** |
| **August., (1996)** | **✔** | **X** | **✔** | **✔** | **X** | **X** |
| **Diniz-de-Figueiredo et al., (2020)** | **✔** | **X** | **✔** | **X** | **✔** | **✔** |
| **Hamid et al., (2018)** | **✔** | **✔** | **X** | **X** | **✔** | **X** |
| **Kielbassa et al., (2017)** | **✔** | **X** | **✔** | **✔** | **X** | **X** |
| **Horiuchi et al., (2016)** | **✔** | **✔** | **X** | **X** | **X** | **X** |
| **Kim et al., (2015)** | **✔** | **X** | **✔** | **X** | **X** | **X** |
| **Iriboz et al., (2014)** | **✔** | **X** | **✔** | **✔** | **X** | **X** |
| **Cohenca et al., (2013)** | **✔** | **X** | **✔** | **✔** | **X** | **X** |
| **Tennert et al., (2013)** | **✔** | **✔** | **X** | **X** | **✔** | **X** |
| **Beus et al., (2012)** | **✔** | **✔** | **✔** | **X** | **X** | **✔** |
| **Zhang et al., (2011)** | **✔** | **X** | **✔** | **✔** | **X** | **X** |
| **Natera et al., (2011)** | **✔** | **X** | **✔** | **✔** | **X** | **X** |
| **Cheng et al., (2011)** | **✔** | **✔** | **X** | **X** | **✔** | **X** |
| **Christiansen et al., (2009)** | **✔** | **X** | **✔** | **✔** | **X** | **X** |
| **Benenati et al., (2002)** | **✔** | **✔** | **✔** | **X** | **✔** | **X** |
| **Kvist., (2000)** | **✔** | **X** | **✔** | **✔** | **✔** | **✔** |
| **Mayhew et al., (1999)** | **✔** | **X** | **X** | **X** | **✔** | **X** |
| **Fava et al., (1994)** | **✔** | **✔** | **X** | **✔** | **X** | **✔** |
| **Smith et al., (1993)** | **✔** | **✔** | **✔** | **X** | **✔** | **✔** |
| **Saunders et al., (1991)** | **✔** | **X** | **✔** | **✔** | **X** | **X** |
| **Karakov et al., (2018)** | **✔** | **X** | **✔** | **X** | **✔** | **✔** |
| **Ortega-Sánchez et al., (2009)** | **✔** | **X** | **✔** | **✔** | **X** | **X** |
| **Marques et al., (2023)** | **✔** | **X** | **✔** | **✔** | **✔** | **✔** |
| **Terauchi et al., (2023)** | **✔** | **X** | **✔** | **✔** | **X** | **X** |
| **Guimarães et al., (2021)** | **✔** | **X** | **✔** | **X** | **X** | **✔** |
| **Barcelos et al., (2012)** | **✔** | **X** | **✔** | **✔** | **X** | **X** |
| **López-Valverde et al., (2023)** | **✔** | **✔** | **✔** | **X** | **✔** | **✔** |
| **Jang et al., (2015)** | **✔** | **X** | **✔** | **✔** | **X** | **X** |
| **Asgary et al., (2014)** | **✔** | **X** | **✔** | **✔** | **X** | **X** |
| **Toia et al., (2022)** | **✔** | **✔** | **✔** | **✔** | **✔** | **X** |
| **Özdemir et al., (2019)** | **✔** | **X** | **✔** | **X** | **X** | **✔** |
| **Rodrigues et al., (2017)** | **✔** | **X** | **X** | **X** | **X** | **X** |
| **Alves., (2010)** | **✔** | **X** | **X** | **X** | **X** | **✔** |
| **Setzer et al., (2011)** | **✔** | **X** | **✔** | **✔** | **✔** | **✔** |
| **Strange et al., (2019)** | **✔** | **✔** | **✔** | **X** | **✔** | **✔** |
| **Dhafar et al., (2022)** | **✔** | **✔** | **✔** | **X** | **✔** | **✔** |
| **Casey et al., (2022)** | **✔** | **✔** | **✔** | **X** | **✔** | **✔** |
| **Zhang et al., (2021)** | **✔** | **X** | **✔** | **✔** | **✔** | **✔** |
| **Williams-Beecher et al., (2023)** | **✔** | **✔** | **✔** | **X** | **✔** | **✔** |
| **Fernández et al., (2017)** | **✔** | **✔** | **✔** | **X** | **✔** | **✔** |
| **Wong et al., (2017)** | **✔** | **X** | **✔** | **✔** | **X** | **X** |
| **Ramey et al., (2017)** | **✔** | **X** | **✔** | **X** | **✔** | **X** |
| **Barborka et al., (2017)** | **✔** | **✔** | **✔** | **X** | **✔** | **✔** |
| **Khalighinejad et al., (2017)** | **✔** | **X** | **✔** | **✔** | **X** | **X** |
| **Silujjai et al., (2017)** | **✔** | **✔** | **✔** | **X** | **✔** | **✔** |
| **Orhan et al., (2017)** | **✔** | **✔** | **✔** | **✔** | **X** | **X** |
| **Guldener et al., (2017)** | **✔** | **✔** | **✔** | **X** | **X** | **X** |
| **Schwendicke et al., (2016)** | **✔** | **X** | **✔** | **✔** | **X** | **X** |
| **Conner et al., (2007)** | **✔** | **X** | **✔** | **✔** | **X** | **X** |
| **Cheung et al., (2009)** | **✔** | **✔** | **X** | **X** | **✔** | **X** |
| **Siqueira et al., (2008)** | **✔** | **✔** | **✔** | **X** | **✔** | **✔** |
| **Fernández et al., (2013)** | **✔** | **✔** | **✔** | **X** | **✔** | **✔** |
| **Ricucci et al., (2011)** | **✔** | **✔** | **✔** | **X** | **✔** | **✔** |
| **Raedel et al., (2015)** | **✔** | **X** | **✔** | **X** | **X** | **X** |
| **Burry et al., (2016)** | **✔** | **X** | **✔** | **X** | **X** | **X** |
| **Sarıyılmaz et al., (2016)** | **✔** | **X** | **X** | **X** | **✔** | **X** |
| **Mindiola et al., (2006)** | **✔** | **X** | **✔** | **X** | **X** | **X** |
| **Nagasiri et al., (2005)** | **✔** | **✔** | **✔** | **X** | **✔** | **X** |
| **Salehrabi et al., (2004)** | **✔** | **X** | **✔** | **X** | **✔** | **✔** |
| **Farzaneh et al., (2004)** | **✔** | **✔** | **✔** | **X** | **✔** | **✔** |
| **Friedman et al., (2003)** | **✔** | **✔** | **✔** | **X** | **✔** | **✔** |
| **Ruiz et al., (2017)** | **✔** | **X** | **✔** | **X** | **✔** | **X** |
| **AlRahabi., (2017)** | **✔** | **X** | **✔** | **✔** | **X** | **X** |
| **Javed et al., (2022)** | **✔** | **✔** | **X** | **X** | **✔** | **X** |
| **Witherspoon et al., (2008)** | **✔** | **✔** | **✔** | **X** | **✔** | **✔** |
| **Kontakiotis et al., (2008)** | **✔** | **✔** | **✔** | **X** | **✔** | **X** |
| **Cusimano et al., (2010)** | **✔** | **X** | **✔** | **✔** | **X** | **X** |
| **Moura et al., (2009)** | **✔** | **X** | **X** | **X** | **✔** | **X** |
| **Liu et al., (2014)** | **✔** | **✔** | **✔** | **✔** | **X** | **✔** |
| **Burns et al., (2023)** | **✔** | **X** | **✔** | **X** | **X** | **X** |
| **He et al., (2017)** | **✔** | **✔** | **✔** | **✔** | **✔** | **✔** |
| **Zanjir et al., (2023)** | **✔** | **X** | **✔** | **X** | **✔** | **✔** |
| **Herbst et al., (2023)** | **✔** | **✔** | **X** | **X** | **✔** | **X** |
| **Bernstein et al., (2012)** | **✔** | **X** | **✔** | **✔** | **✔** | **✔** |
| **Yee et al., (2018)** | **✔** | **X** | **✔** | **X** | **✔** | **✔** |
| **Alghofaily et al., (2018)** | **✔** | **✔** | **✔** | **X** | **✔** | **X** |
| **Sjogren et al., (1990)** | **✔** | **X** | **✔** | **X** | **✔** | **✔** |
| **Cheung., (2002)** | **✔** | **X** | **✔** | **✔** | **✔** | **✔** |
| **Lazarski et al., (2001)** | **✔** | **X** | **✔** | **X** | **X** | **✔** |
| **Zandi et al., (2016)** | **✔** | **✔** | **X** | **X** | **X** | **X** |
| **Rana et al., (2022)** | **✔** | **✔** | **X** | **X** | **✔** | **✔** |
| **de-Figueiredo et al., (2020)** | **✔** | **✔** | **✔** | **X** | **✔** | **✔** |
| **de Castro Rizzi-Maia et al., (2016)** | **✔** | **X** | **✔** | **✔** | **X** | **X** |
| **Yan et al., (2022)** | **✔** | **X** | **✔** | **✔** | **X** | **X** |
| **Zamparini et al., (2021)** | **✔** | **✔** | **✔** | **✔** | **✔** | **✔** |
| **Fracassi et al., (2013)** | **✔** | **✔** | **X** | **X** | **✔** | **✔** |
| **Tolibah et al., (2022)** | **✔** | **✔** | **✔** | **✔** | **✔** | **✔** |
| **de Miranda et al., (2018)** | **✔** | **✔** | **✔** | **✔** | **✔** | **✔** |
| **Makanjuola et al., (2018)** | **✔** | **X** | **✔** | **✔** | **✔** | **X** |

**Supplementary Table 1.** List of studies excluded after full-text assessment, with reasons for exclusion (ineligible study design, insufficient follow-up, or incomplete outcome data).

|  | **Beta** | **SE** | **p-value** | **95%CI Beta** | **R^2^** |
| --- | --- | --- | --- | --- | --- |
| **MATERIAL** |  |  | **0.030*** |  | 6.08% |
| MTA vs. GP (ref.) | 0.084 | 0.043 | 0.052 | -0.001 0.169 |  |
| CH vs. GP (ref.) | 0.099 | 0.054 | 0.066 | -0.007 0.206 |  |
| CH vs. MTA (ref.) | 0.031 | 0.063 | 0.621 | -0.093 0.156 |  |
|  |  |  |  |  |  |
| **SEALER** |  |  | 0.373 |  | 1.24% |
| BIO vs. AHP (ref.) | 0.018 | 0.039 | 0.642 | -0.058 0.095 |  |
| CH vs. AHP (ref.) | 0.001 | 0.040 | 0.973 | -0.076 0.079 |  |
| RES vs. AHP (ref.) | -0.003 | 0.032 | 0.933 | -0.065 0.059 |  |
| GLA vs. AHP (ref.) | 0.070 | 0.066 | 0.284 | -0.058 0.199 |  |
| ZNC vs. AHP (ref.) | -0.048 | 0.028 | 0.091 | -0.104 0.007 |  |
| CH vs. BIO (ref.) | -0.017 | 0.051 | 0.739 | -0.116 0.082 |  |
| RES vs. BIO (ref.) | -0.021 | 0.045 | 0.640 | -0.108 0.067 |  |
| GLA vs. BIO (ref.) | 0.052 | 0.073 | 0.474 | -0.091 0.195 |  |
| ZNC vs. BIO (ref.) | -0.066 | 0.043 | 0.118 | -0.150 0.017 |  |
| RES vs. CH (ref.) | -0.004 | 0.045 | 0.929 | -0.092 0.084 |  |
| GLA vs. CH (ref.) | 0.069 | 0.073 | 0.345 | -0.074 0.213 |  |
| ZNC vs. CH (ref.) | -0.050 | 0.042 | 0.249 | -0.134 0.035 |  |
| GLA vs. RES (ref.) | 0.073 | 0.069 | 0.291 | -0.063 0.209 |  |
| ZNC vs. RES (ref.) | -0.046 | 0.036 | 0.204 | -0.116 0.025 |  |
| ZNC vs. GLA (ref.) | -0.119 | 0.068 | 0.080 | -0.251 0.014 |  |
|  |  |  |  |  |  |
| **AGE** | -0.003 | 0.001 | **0.001**** | -0.004 -0.001 | 10.8% |
|  |  |  |  |  |  |
| **GENDER (% Male)** | -0.001 | 0.001 | 0.287 | -0.003 0.001 | 0.75% |
|  |  |  |  |  |  |
| **PRE PULPAL DIAG** |  |  |  |  |  |
| Irreversible (%) | 0.001 | 0.000 | 0.230 | -0.000 0.001 | 0.00% |
| Necrotic (%) | 0.001 | 0.000 | 0.128 | -0.000 0.001 | 2.03% |
| Prev. treated (%) | -0.001 | 0.000 | **0.005**** | -0.002 -0.001 | 11.2% |
|  |  |  |  |  |  |
| **PRE PERIAP DIAG** |  |  |  |  |  |
| Normal (%) | 0.001 | 0.003 | 0.672 | -0.005 0.007 | 0.00% |
| Symptomatic AP (%) | -0.001 | 0.001 | 0.457 | -0.002 0.001 | 0.00% |
| Asymptomatic AP (%) | 0.001 | 0.001 | 0.885 | -0.001 0.001 | 0.00% |
| Acute apical abscess (%) | 0.006 | 0.004 | 0.101 | -0.001 0.013 | 5.21% |
| Chronic apical abscess (%) | 0.002 | 0.001 | 0.798 | -0.001 0.002 | 0.00% |
|  |  |  |  |  |  |
| **PRE OPERATIVE PAIN (%)** | -0.001 | 0.001 | 0.136 | -0.003 0.000 | 9.16% |
| **POST OPERATIVE PAIN (%)** | 0.001 | 0.001 | 0.599 | -0.002 0.003 | 0.00% |
|  |  |  |  |  |  |
| **INSTRUMENTATION TECHNIQUE** |  |  | 0.472 |  | 0.00% |
| Rotary vs. Manual (ref.) | 0.013 | 0.023 | 0.586 | -0.033 0.058 |  |
| Reciproc vs. Manual (ref.) | 0.047 | 0.039 | 0.221 | -0.028 0.123 |  |
| Reciproc vs. Rotary (ref.) | 0.035 | 0.036 | 0.332 | -0.035 0.105 |  |
|  |  |  |  |  |  |
| **IRRIGATION** |  |  |  |  |  |
| NaOCl+EDTA vs. NaOCl (ref.) | -0.016 | 0.020 | 0.431 | -0.056 0.024 | 0.11% |
|  |  |  |  |  |  |
| **RX EVALUATION** |  |  | 0.292 |  | 1.57% |
| CBCT vs. PA (ref.) | -0.049 | 0.047 | 0.292 | -0.142 0.043 |  |
| Both vs. PA (ref.) | 0.060 | 0.053 | 0.262 | -0.045 0.165 |  |
| Both vs. CBCT (ref.) | 0.109 | 0.070 | 0.118 | -0.028 0.247 |  |
|  |  |  |  |  |  |
| **ARCH (% in maxilla)** | 0.001 | 0.000 | **0.006**** | 0.000 0.002 | 14.7% |
|  |  |  |  |  |  |
| **TOOTH TYPE** |  |  |  |  |  |
| Anterior (%) | 0.000 | 0.000 | 0.454 | -0.001 0.001 | 0.00% |
| Premolar (%) | 0.000 | 0.000 | 0.960 | -0.001 0.001 | 0.00% |
| Molar (%) | 0.000 | 0.000 | 0.494 | -0.001 0.001 | 0.00% |
|  |  |  |  |  |  |
| **OPERATOR** |  |  | **<0.001***** |  | 33.4% |
| Post-Grad. vs. Under-Grad. (ref.) | 0.048 | 0.052 | 0.363 | -0.055 0.150 |  |
| GP vs. Under-Grad. (ref.) | 0.037 | 0.059 | 0.526 | -0.078 0.152 |  |
| Specialist vs. Under-Grad. (ref.) | 0.137 | 0.050 | **0.006**** | 0.039 0.235 |  |
| GP vs. Post-Grad. (ref.) | -0.010 | 0.038 | 0.786 | -0.086 0.065 |  |
| Specialist vs. Post-Grad. (ref.) | 0.089 | 0.023 | **<0.001***** | 0.044 0.135 |  |
| GP vs. Specialist (ref.) | -0.099 | 0.035 | **0.005**** | -0.169 -0.031 |  |
|  |  |  |  |  |  |
| **SINGLE VISIT (%)** | 0.000 | 0.000 | 0.440 | -0.000 0.001 | 0.00% |
|  |  |  |  |  |  |
| **TYPE OF TREATMENT** |  |  |  |  |  |
| Re-RCT vs. Primary (ref.) | -0.073 | 0.021 | **0.001**** | -0.115 -0.031 | 7.36% |

^*p<0.05; **p<0.01; ***p<0.001^

**Supplementary Table 2.** Results of simple meta-regression models for success rate by independent variables.


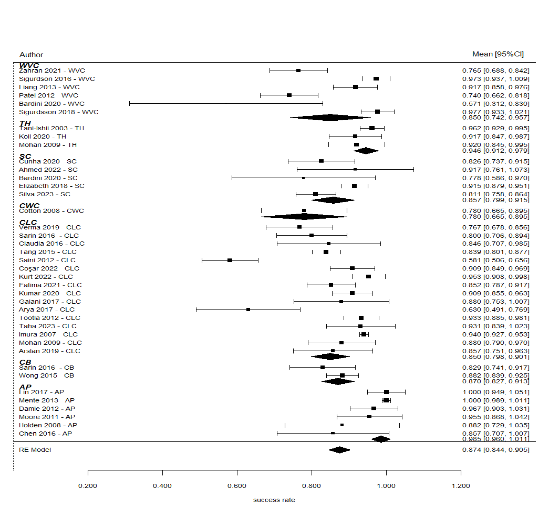

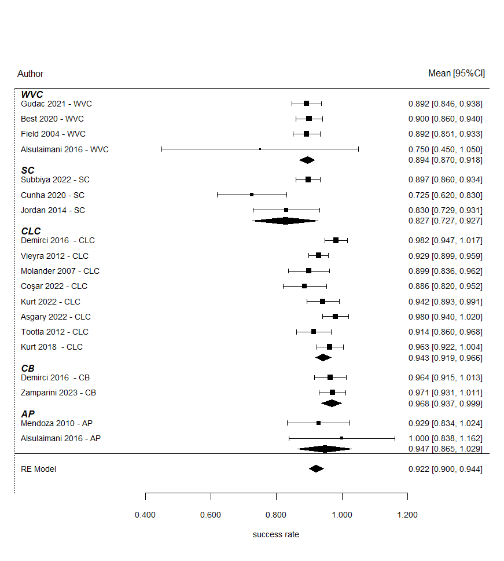

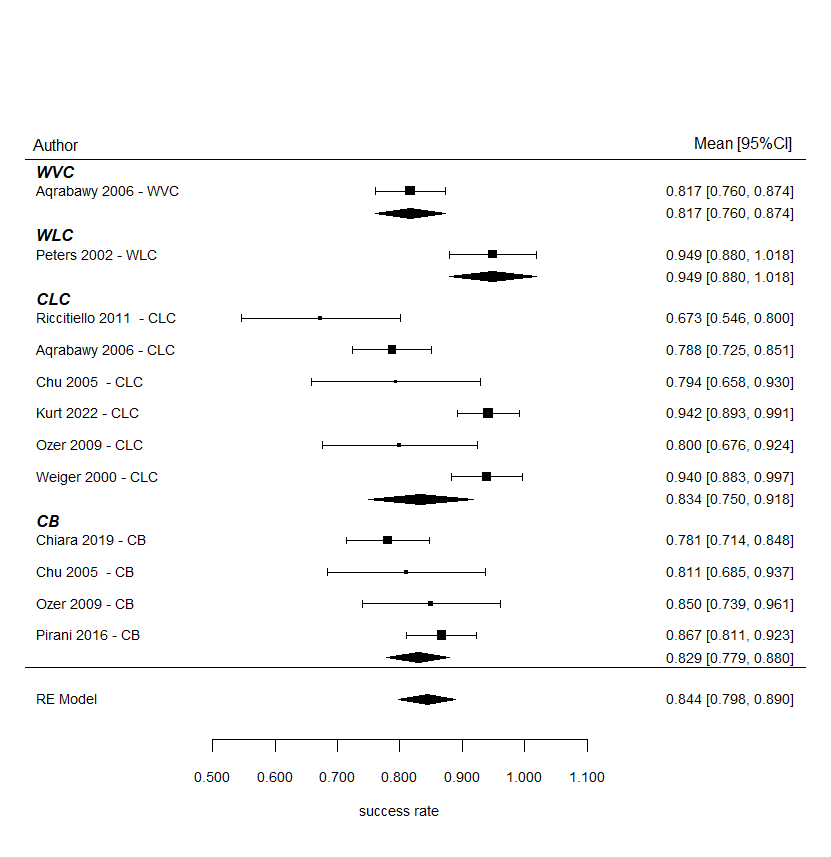

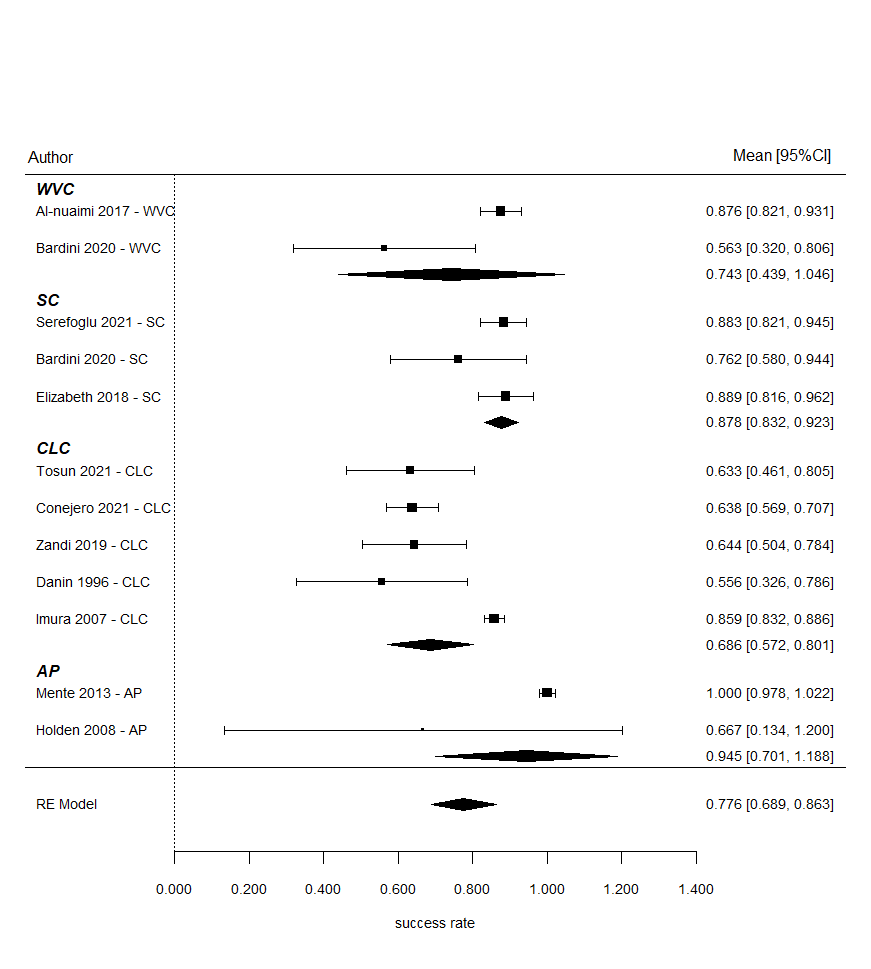

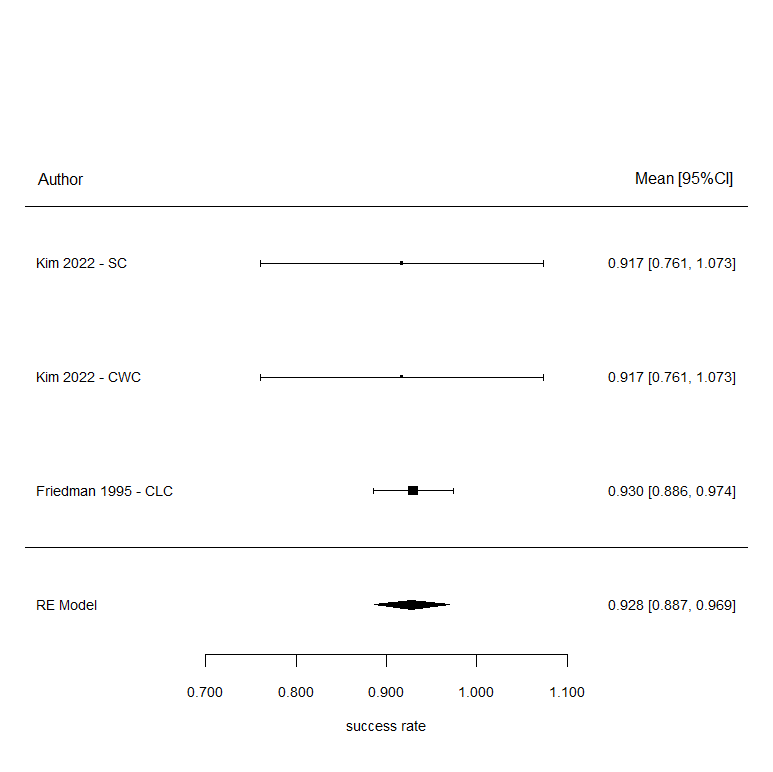

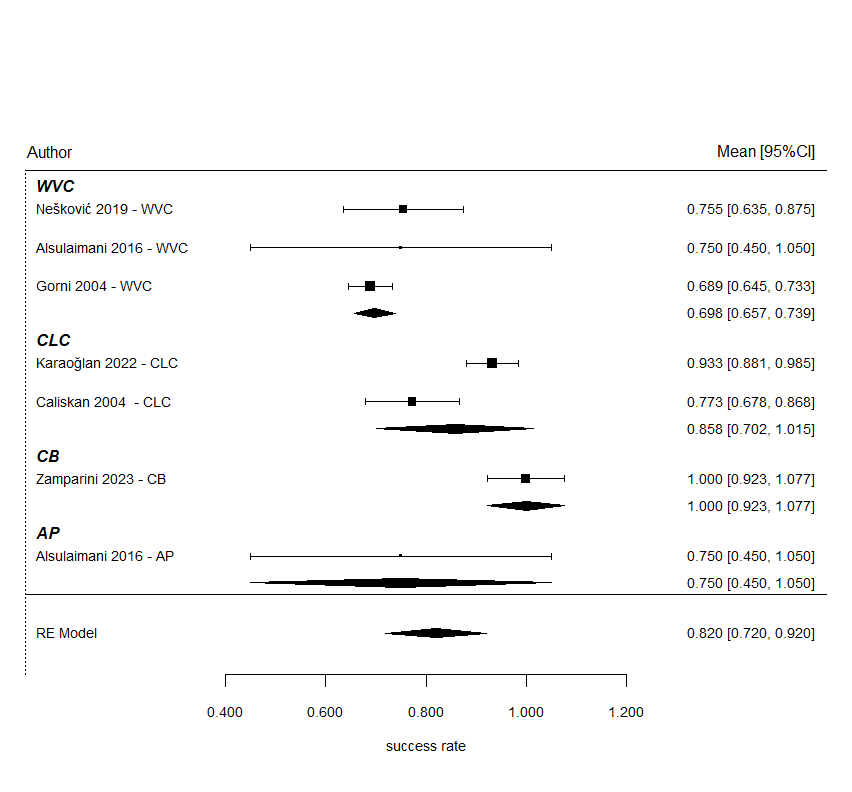

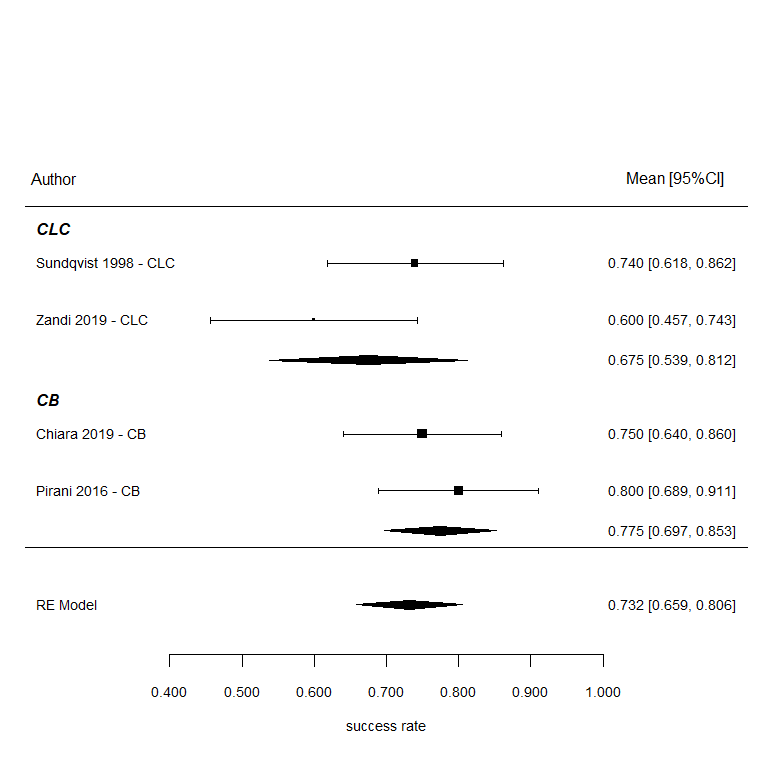

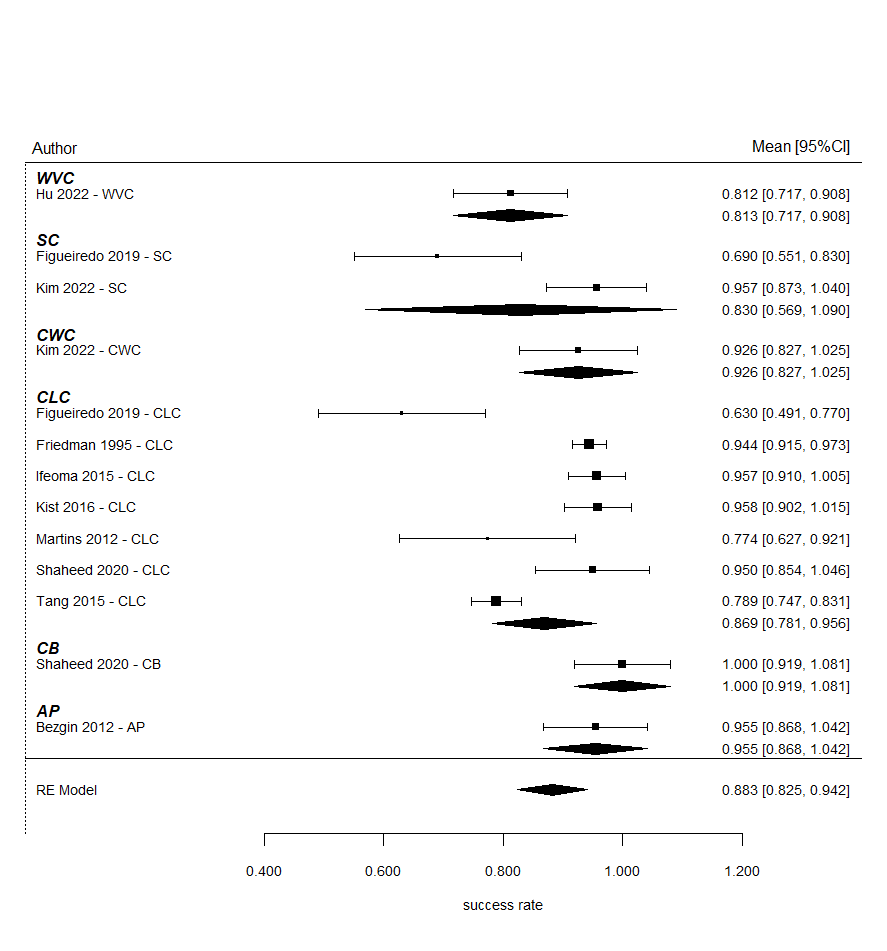


**f**

**e**

**d**

**c**

**a**

**b**

**h**

**g**

**Supplementary Figure 1**. Forest plots present a comparative analysis of healing outcomes following two endodontic procedures, where the AP technique was included in the analysis. The forest plots depict results for primary root canal treatments at (a) 6-month, (b) 12-month, (c) 24-month, and (d) 3+ year follow-up points. Correspondingly, outcomes for root canal retreatments are shown for the same periods at (e) 6 months, (f) 12 months, (g) 24 months, and (h) 3+ years.
